# Monopolin Subunit Csm1 Associates with MIND Complex to Establish Monopolar Attachment of Sister Kinetochores at Meiosis I

**DOI:** 10.1371/journal.pgen.1003610

**Published:** 2013-07-04

**Authors:** Sourav Sarkar, Rajesh T. Shenoy, Jacob Z. Dalgaard, Louise Newnham, Eva Hoffmann, Jonathan B. A. Millar, Prakash Arumugam

**Affiliations:** 1 University of Warwick, Coventry, United Kingdom; 2 MRC Genome Damage and Stability Centre, School of Life Sciences, University of Sussex, Brighton, United Kingdom; Fred Hutchinson Cancer Research Center, United States of America

## Abstract

Sexually reproducing organisms halve their cellular ploidy during gametogenesis by undergoing a specialized form of cell division known as meiosis. During meiosis, a single round of DNA replication is followed by two rounds of nuclear divisions (referred to as meiosis I and II). While sister kinetochores bind to microtubules emanating from opposite spindle poles during mitosis, they bind to microtubules originating from the same spindle pole during meiosis I. This phenomenon is referred to as mono-orientation and is essential for setting up the reductional mode of chromosome segregation during meiosis I. In budding yeast, mono-orientation depends on a four component protein complex referred to as monopolin which consists of two nucleolar proteins Csm1 and Lrs4, meiosis-specific protein Mam1 of unknown function and casein kinase Hrr25. Monopolin complex binds to kinetochores during meiosis I and prevents bipolar attachments. Although monopolin associates with kinetochores during meiosis I, its binding site(s) on the kinetochore is not known and its mechanism of action has not been established. By carrying out an imaging-based screen we have found that the MIND complex, a component of the central kinetochore, is required for monopolin association with kinetochores during meiosis. Furthermore, we demonstrate that interaction of monopolin subunit Csm1 with the N-terminal domain of MIND complex subunit Dsn1, is essential for both the association of monopolin with kinetochores and for monopolar attachment of sister kinetochores during meiosis I. As such this provides the first functional evidence for a monopolin-binding site at the kinetochore.

## Author Summary

All sexually reproducing organisms produce haploid gametes from diploid cells *via* meiosis. During meiosis, one round of DNA replication is followed by two rounds of nuclear division (called meiosis I and II). This is unlike mitotically proliferating cells wherein one round of DNA replication is followed by one round of nuclear division. During meiosis I, sister chromatids move towards the same spindle pole unlike in mitosis where they move towards opposite spindle poles. Poleward chromosome movement is achieved by association of kinetochores (a complex network of proteins assembled at centromeres on chromosomes) with microtubule ends emanating from spindle poles. The basis of the contrasting fate of sister chromatids during mitosis and meiosis I is best studied in budding yeast in which a protein complex called monopolin binds to sister kinetochores during meiosis I and ensures that they face the same spindle pole. But precisely how monopolin binds to kinetochores was unknown. In this study, we have identified a monopolin's receptor at the kinetochore. Disabling the receptor did not affect mitotic growth but severely compromised meiotic chromosome segregation. Cells lacking the monopolin receptor attempt to segregate sister chromatids towards opposite spindle poles during meiosis I with catastrophic genetic consequences.

## Introduction

Meiosis is a specialized form of cell division that results in the formation of haploid gametes from diploid cells. Two nuclear divisions following one round of DNA replication results in halving of ploidy during meiosis. Four innovations during meiosis allow cells to achieve this remarkable step [1], [2]. Firstly, recombination between homologs results in covalent connections between them, which are cytologically manifested as chiasmata. This is required for bi-orientation of homologs during meiosis I. Secondly, sister kinetochores mono-orient during meiosis I namely that they bind to microtubules emanating from the same spindle pole. Thirdly, centromeric cohesion is protected from separase cleavage during meiosis I. Centromeric cohesion is required for bi-orientation of sister centromeres during meiosis II. Fourthly, a second round of DNA replication is prevented between the two meiotic divisions. Understanding how meiotic cell cycle works is crucial for understanding the molecular basis of infertility, spontaneous abortions and aneuploidy-related disorders such as Down syndrome in humans.

Monopolar attachment of sister kinetochores is essential for setting up the reductional mode of chromosome segregation during meiosis I. During mitosis, sister kinetochores bind to microtubules from opposite spindle poles, a process referred to as bi-orientation. During meiosis I, homologs connected by chiasmata bi-orient on the meiosis I spindle. Tension created by sister chromatid cohesion distal to chiasmata stabilizes the bi-oriented state. For homologs to segregate towards opposite spindle poles, it is essential that sister kinetochores bind to microtubules originating from the same spindle pole.

Research over the last twelve years has shown that monopolar attachment in budding yeast is mediated by the ‘monopolin’ complex , which is composed of the Csm1, Lrs4, Mam1 and Hrr25 proteins [3]–[5]. Csm1 and Lrs4 are nucleolar proteins expressed during the mitotic cell cycle. They are required for rDNA silencing and for preventing unequal sister chromatid exchange at the rDNA repeats [6]. Csm1 and Lrs4 interact with Tof2 which binds to rDNA *via* interaction with the RENT (*r*egulator of *n*ucleolar silencing and *t*elophase) complex composed of Net1, Cdc14 and Sir2 [6]. However during meiosis I, Csm1 and Lrs4 are released from the nucleolus [4] and this requires the activity of polo-like kinase Cdc5 [7]. Csm1 and Lrs4 associate with meiosis-specific protein Mam1 and casein kinase-1 Hrr25 to form the monopolin complex which binds to kinetochores. The kinase activity of Hrr25 is required for monopolar attachment but not for monopolin binding to kinetochores [5]. Following nucleolar release Lrs4 is hyperphosphorylated by Dbf4-dependent kinase Cdc7 and Cdc5 in league with a meiosis-specific protein called Spo13 [8]. Hyperphosphorylation of Lrs4 helps association of monopolin with kinetochores.

Monopolin complex was suggested to work by crosslinking the sister kinetochores together about ten years ago [4]. Crystal structure and electron microscopic analysis of Csm1/Lrs4 complex indicated that it forms a V-shaped structure with 2 kinetochore binding globular domains of Csm1 separated by 10 nm [9]. It was therefore proposed that monopolins could crosslink the sister kinetochores *via* the 2 globular kinetochore-binding domains such that they face the same spindle pole. Recently, the structure of a fragment of Mam1 bound to Csm1 was determined which shows that Mam1 wraps around the globular domain of the Csm1 dimer [10].

Homologs of monopolin subunits Csm1, Lrs4 and Mam1 exist only in some fungi. In fission yeast Pcs1 and Mde4 are homologs of Csm1 and Lrs4 respectively. Interestingly, Pcs1 and Mde4 are not required for monopolar attachment during meiosis I but are necessary to prevent merotelic attachments (where a single kinetochore binds to microtubules from both spindle poles) during mitosis and meiosis II [4], [11], [12]. The fission yeast kinetochore has 2–4 MT binding sites compared to just one site for the budding yeast kinetochore. An attractive hypothesis was proposed to explain the contrasting phenotypes of monopolin mutants in budding and fission yeasts [4]. While monopolins were proposed to clamp microtubule binding sites from two sister kinetochores in budding yeast, they crosslink two adjacent microtubule binding sites from the same sister kinetochore in fission yeast. It was recently reported that Pcs1 and Mde4 prevent merotelic attachment in fission yeast by targeting condensin complex to the kinetochores [13].

Determining how monopolin associates with the kinetochore is essential for elucidating the mechanism of monopolar attachment. The budding yeast kinetochore is made up of several multi-subunit protein complexes that assemble on centromeres and help in the segregation of chromosomes towards spindle poles [14], [15]. The kinetochore can be classified into three layers namely inner, central and outer. While the inner kinetochore directly interacts with centromeric DNA, the outer kinetochore interacts with the microtubule ends. The inner and outer layers are connected by the central kinetochore. The monopolin subunit Csm1 has been found to interact with MIND complex (a part of the central kinetochore composed of Mtw1, Nsl1, Nnf1 and Dsn1 proteins) and CENP-C homolog Mif2 (a part of the inner kinetochore) *in vitro* [9]. Replacement of amino acid residues in a conserved hydrophobic loop of Csm1 blocks its interaction with Dsn1 and Mif2 *in vitro* and prevents monopolar attachment during meiosis I [9]. However the amino acid replacements also prevent interaction of Csm1 with the nucleolar protein Tof2 and affect rDNA silencing [9] suggesting that they might perturb the overall structure of Csm1. Moreover, the interaction of Csm1 with Mif2 has been recently questioned [10]. Csm1 has also been shown to interact with Ctf19, a non-essential central kinetochore protein and the MIND complex subunit Dsn1 in a high throughput yeast 2-hybrid screen [16]. However the functional significance of these interactions has not been analysed. By carrying out an imaging based screen, we show that the MIND complex is required for stable association of monopolins with kinetochores during meiosis I. By coupling targeted mutagenesis to a binding assay, we have identified a ‘Csm1-interaction domain’ (CID) in Dsn1. Deleting CID had no effect on mitotic growth but severely compromised meiotic chromosome segregation. Furthermore we show that cells lacking the CID do not localize monopolins to the kinetochore and attempt to bi-orient sister kinetochores on the meiosis I spindle. Our work provides a mechanism for monopolar attachment during meiosis I in budding yeast.

## Results

### The MIND complex and CENP-C homolog Mif2 are required for monopolin association with kinetochores

**Figure 1:**
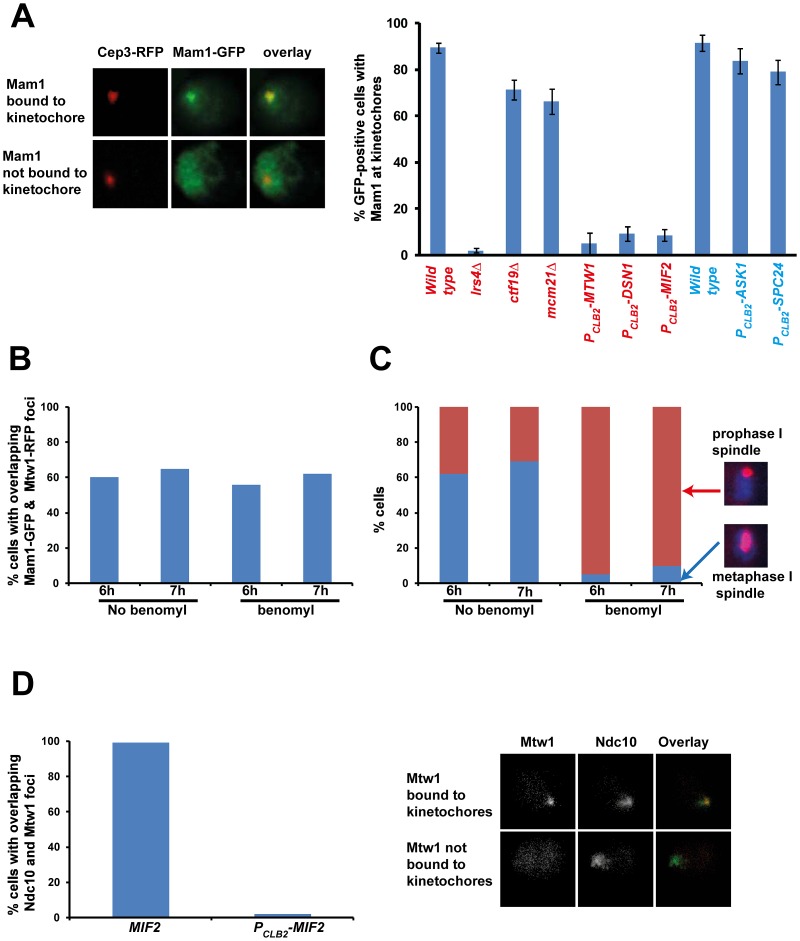
Monopolin localization to kinetochores requires the MIND complex and CENP-C. *A) P_CLB2_-CDC20 CEP3-RFP MAM1-GFP* or *P_CLB2_-CDC20 MTW1-RFP MAM1-GFP* cells and genotypically identical cells but containing either *lrs4Δ* or *ctf19Δ* or *mcm21Δ* or expressing *P_CLB2_-MTW1*, *P_CLB2_-DSN1*, *P_CLB2_-MIF2*, *P_CLB2_-ASK1* or *P_CLB2_-SPC24* were induced to enter meiosis. Co-localization of Mam1-GFP with either Mtw1-RFP or Cep3-RFP signals was assayed after 5–8 hours in SPM. Representative image of cells with either Mam1 localized to kinetochores or where Mam1 is distributed throughout the nucleus is shown on the left. The average number of GFP-positive cells in which Mam1 co-localises with the kinetochore marker (with Cep3 in red and Mtw1 in blue) was assessed from 3 independent cultures (300 cells per experiment). B) *MTW1-RFP MAM1-GFP P_CLB2_-CDC20* cells were induced to enter meiosis by transferring them to SPM. After 4 hours the cultures were split into two and either benomyl (120 µg/ml) or DMSO was added to the cultures. The percentage of GFP-positive cells showing overlapping Mtw1-RFP and Mam1-GFP signals is shown in the plot. C) *In situ* immunofluorescence of cells from 1B was performed and cells were stained with anti-tubulin antibodies and DAPI. Percentage of cells with metaphase I/prophase I-like spindles is indicated. D) *NDC10-GFP MTW1-RFP P_CLB2_-CDC20* strains containing either *MIF2* or *P_CLB2_-MIF2* were induced to undergo meiosis by transferring them to SPM. After 5 hours, cells were fixed and analysed by fluorescence microscopy. The percentage of cells with overlapping Mtw1 and Ndc10 foci are shown. Sample images of cells with Mtw1 bound to kinetochores and not bound to kinetochores are shown. Data shown in panels B–D are of single representative experiment of at least three independent repeats.

To identify the binding sites of monopolin at the kinetochore, we first established an assay for detecting association of monopolins to the kinetochore during meiosis I. To do this we attached Green Fluorescent Protein (GFP) to Mam1 (to visualize monopolins) and Red Fluorescent Protein (RFP) to Cep3/Mtw1 (to visualize kinetochores). To arrest cells in metaphase I (which increases the proportion of cells with Mam1 at kinetochores), we replaced the promoter of *CDC20* (which encodes an activator of Anaphase-Promoting Complex) with the mitosis-specific promoter *P_CLB2_* [17]. We find that Mam1 co-localizes with Cep3 at the kinetochore in 90% of GFP-positive *MAM1-GFP CEP3-RFP P_CLB2_-CDC20* cells following 7 hours of incubation in sporulation medium (SPM) (Figure 1A). Co-localisation of Mam1 and Cep3/Mtw1 was dependent on Lrs4 (Figure 1A and Table 1), confirming that binding of monopolin subunits to the kinetochore is inter-dependent [4]. Importantly, addition of benomyl abolished formation of metaphase I spindles but did not affect association of Mam1 with kinetochores (Figure 1B and 1C), indicating that binding of monopolin to kinetochores is not dependent on kinetochore-microtubule interaction.

**Table 1:** Localization of Mam1 to kinetochores during meiosis I in wild type and various mutant strains.

Strain genotype	Percentage of GFP-positive cells	Percentage of GFP-positive cells with coinciding Mam1-GFP and Cep3-/Mtw1- RFP foci Mean±SD
*Wild type*	58	89±2
*lrs4Δ*	60	1±1
*ctf19Δ*	62	71±4
*mcm21Δ*	49	66±5
*ctf3Δ*	54	84±4
*mcm22Δ*	50	78±8
*nkp1Δ*	70	91±4
*nkp2Δ*	67	86±4
*mcm16Δ*	49	76±5
*iml3Δ*	65	85±5
*chl4Δ*	57	80±4
*P_CLB2_-MTW1*	59	9±3
*P_CLB2_-DSN1*	51	8±2
*P_CLB2_-MIF2*	56	5±4
***Wild type***	65	91±4
***lrs4Δ***	58	2±1
***P_CLB2_-SPC24***	59	79±5
***P_CLB2_-ASK1***	63	84±5

Data obtained from the microscopic screen are presented above. A hundred cells in triplicate were scored for each strain after transfer to SPM for 5–8 h. The average percentage of cells showing Mam1-GFP expression and percentage of GFP-positive cells having co-localization of Mam1-GFP foci with Cep3–RFP or Mtw1-RFP are indicated. Genotypes of Cep3-RFP and Mtw1-RFP strains are indicated in normal type and boldface respectively.

To determine which kinetochore protein(s) are required for monopolin complex recruitment, we inactivated 14 genes encoding kinetochore proteins either by gene deletion (for non-essential genes) or meiosis-specific suppression of gene expression by replacement of native promoters with *P_CLB2_* (for essential genes). We find that inactivation of Ctf19 and other components of the Ctf19 complex including Mcm21, Chl4, Ctf3, Mcm22, Nkp1, Nkp2, Mcm16 and Iml3 had little or no effect on association of monopolin with kinetochores (Table 1, Figure 1A). These results are consistent with the observation that about 80% of *ctf19Δ* cells segregate chromosomes reductionally during meiosis I [18]. Similarly, inactivation of Spc24 and Ask1, which are components of the Ndc80 and Dam1 complexes respectively, did not affect Mam1 binding to kinetochores (Table 1, Figures 1A). We confirmed that both Spc24 and Ask1 were efficiently depleted in *P_CLB2_-SPC24* and *P_CLB2_-ASK1* strains following transfer to SPM (Figure S1). This suggests that monopolin recruitment to kinetochores does not require the outer kinetochore components. In contrast, inactivation of MIND complex components Mtw1 and Dsn1 and the CENP-C homolog Mif2 severely affected association of monopolin with kinetochores (Table 1 and Figure 1A). We note that depletion of Mif2 during meiosis also affected recruitment of MIND complex to kinetochores (Figure 1D). This is consistent with the observations that CENP-C is required for stable association of the Mis12 (counterpart of MIND) complex with kinetochores in both human and fly cells [19], [20]. These results suggest that the MIND complex is required for monopolin association to the kinetochore.

### The N-terminal domain of Dsn1 interacts with Csm1

**Figure 2:**
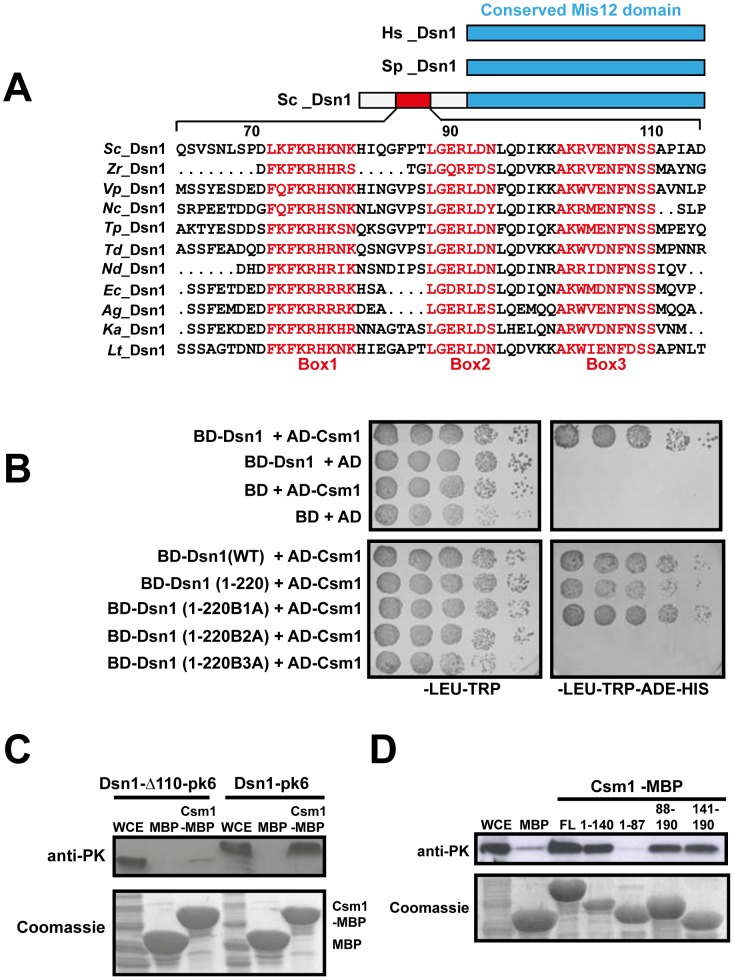
Csm1 interacts with the N-terminal domain of Dsn1. A) Multiple alignment of the amino acid sequences of the N-terminus of Dsn1 from several members of *Saccharomycetes* class of yeasts (*Sc*- *Saccharomyces cerevisiae*; *Zr*-*Zygosaccharomyces rouxii*; *Vp*-*Vanderwaltozyma polyspora*; *Nc*- *Naumovozyma Castelli*; *Tp*- *Tetrapisispora phaffia*; *Nd*- *Naumovozyma dairenensi; Ec*- *Eremothecium cymbala*; *Td*- *Torulaspora delbrueckii*; *Ag*- *Ashbya gossypii*; *Lt*- *Lachancea thermotolerans*; *Ka*- *Kazachatania africana*). Three conserved elements are highlighted (Boxes 1–3). Note that the N-terminal domain is absent in Dsn1 homologs of *S. pombe* and humans. B) Dsn1 interacts with Csm1 in the 2-hybrid assay (Top panel). Transformants of AH109 strain containing pGBKT7-Dsn1 + pGAD-Csm1 or pGBKT7 + pGAD-Csm1 or pGBKT7-Dsn1 + pGAD or pGBKT7 + pGAD were spotted at different dilutions on non-selective SD-LEU-TRP plates and selective SD-LEU-TRP-ADE-HIS plates. Growth was monitored after incubation at 30°C for 2–3 days. Boxes 2 and 3 but not Box 1 in Dsn1 is required for interaction with Csm1 (Bottom panel). Transformants of AH109 strain containing pGBKT7-Dsn1 + pGAD-Csm1 or pGBKT7-Dsn1 (1-220) + pGAD-Csm1 or pGBKT7-Dsn1 (1-220B1A –Box1 alanine substituted) + pGAD-Csm1 or pGBKT7-Dsn1 (1-220B2A) + pGAD-Csm1 or pGBKT7-Dsn1 (1-220B3A) + pGAD-Csm1 were analysed by spot assay as described above. C) Csm1 interacts with N-terminal domain of Dsn1 *in vitro*. Csm1-MBP or MBP was incubated with extracts of yeast cells expressing Dsn1-pk6 or Dsn1-Δ110-pk6, respectively. The proteins bound to the Csm1-MBP beads and MBP beads were subjected to SDS-PAGE followed by either Western analysis using anti-PK antibodies or Coomassie staining. D) Dsn1 interacts with the C-terminal domain of Csm1 *in vitro*. A binding assay was performed by incubating different variants of Csm1 coupled to beads with yeast extracts from cells expressing Dsn1-pk6 and the samples were analysed as described in 2C. FL denotes the full length version of Csm1 (1–190). Data shown (panels B–D) are of single representative experiment of at least three independent repeats.

The hypothesis that the MIND complex directly recruits monopolin to the kinetochore makes three key predictions. Firstly, at least one of its subunits should contain a monopolin-interacting domain. Secondly, mutation of this domain should not affect mitotic chromosome segregation. Thirdly, mutation of the domain however should prevent monopolin binding to kinetochores and monopolar attachment during meiosis I. The MIND complex subunit Dsn1 has been reported to interact with Csm1 in a 2-hybrid assay [16]. We first confirmed the 2-hybrid interaction between full length Csm1 and Dsn1 (Figure 2B Top panel). We then used deletion mutagenesis to map the domain in Dsn1 that interacts with Csm1 (Table 2). While Dsn1 (1–352) interacted with Csm1 as efficiently as full length Dsn1 (1–576), Dsn1 (353–576) did not. We further narrowed down the interaction domain to the first 220 residues of Dsn1 (Table 2 and Figure 2B). However neither Dsn1 (1–130) nor Dsn1 (131–220) interacted with Csm1 suggesting that the N-terminal 220 residues of Dsn1 represents a minimal region sufficient for binding Csm1 (Table 2).

**Table 2:** Mapping the Dsn1 domain that interacts with Csm1 by 2-hybrid analysis.

A.a. residues of Dsn1 fused to Gal4-DBD	Y2H interaction with Csm1
1–576	+
1–352	+
353–576	−
1–220	+
1–130	−
131–220	−

Interaction between the different domains of Dsn1 (fused to Gal4-DBD) and Csm1 (fused to Gal4-AD) was measured by 2-hybrid assays and the results obtained are tabulated above.

To identify motifs crucial for Csm1-Dsn1 interaction, we scrutinized the N-terminal 220 residues of *Saccharomyces cerevisiae* (*S.c*.) Dsn1. The N-terminal domain is conserved amongst the *Saccharomycetes* class of ascomycetes but absent in *Schizosaccharomyces pombe* and human Dsn1 (Figure 2A). A multiple sequence alignment of the N-terminal domain of Dsn1 identified a highly conserved stretch between residues 70–110 in *S.c.* Dsn1 that contains three conserved elements, which we termed Box 1, 2 and 3 (Figure 2A). We find that mutation to alanine of Box 2 and Box 3, but not Box 1, completely abolished Csm1-Dsn1 interaction (Figure 2B Bottom panel). To test whether the N-terminal domain of Dsn1 interacts with Csm1 *in vitro*, we incubated yeast extracts containing either full length Dsn1 or a version lacking the first 110 residues (Dsn1-Δ110), with beads coupled to recombinant Csm1. Full length Dsn1 interacted with Csm1-coated beads, but not to control beads, whereas Dsn1-Δ110 failed to interact with either Csm1-coated or control beads (Figure 2C). This indicates that the first 110 residues of Dsn1 are required for interaction with Csm1. We refer to this region as the Csm1-Interaction Domain (CID). To determine the reciprocal Dsn1-interacting domain in Csm1, we tested the ability of different segments of Csm1 to interact with Dsn1 in an *in vitro* binding assay. Crystal structure of Csm1 indicates that the first N-terminal 82 residues form a coiled-coil domain and the C-terminal residues 83–181 form a globular domain [9]. While regions of Csm1 encoding residues 88–190, 141–190 and 1–140 interacted with Dsn1, the N-terminal 87 residues did not (Figure 2D). This suggests that the globular, but not coiled-coiled, domain of Csm1 interacts with Dsn1. This is consistent with the finding that point mutations that disrupt a conserved hydrophobic patch in globular domain of Csm1 abolished its interaction with Dsn1 *in vitro* [9]. Since Corbett *et al.* [9] used purified Csm1 and Dsn1 proteins in their *in vitro* binding assays, it is quite likely that Csm1 directly interacts with Dsn1 *in vivo*.

### *dsn1-Δ110* suppresses the poor spore viability phenotype of *spo11Δ spo12Δ* cells

**Figure 3:**
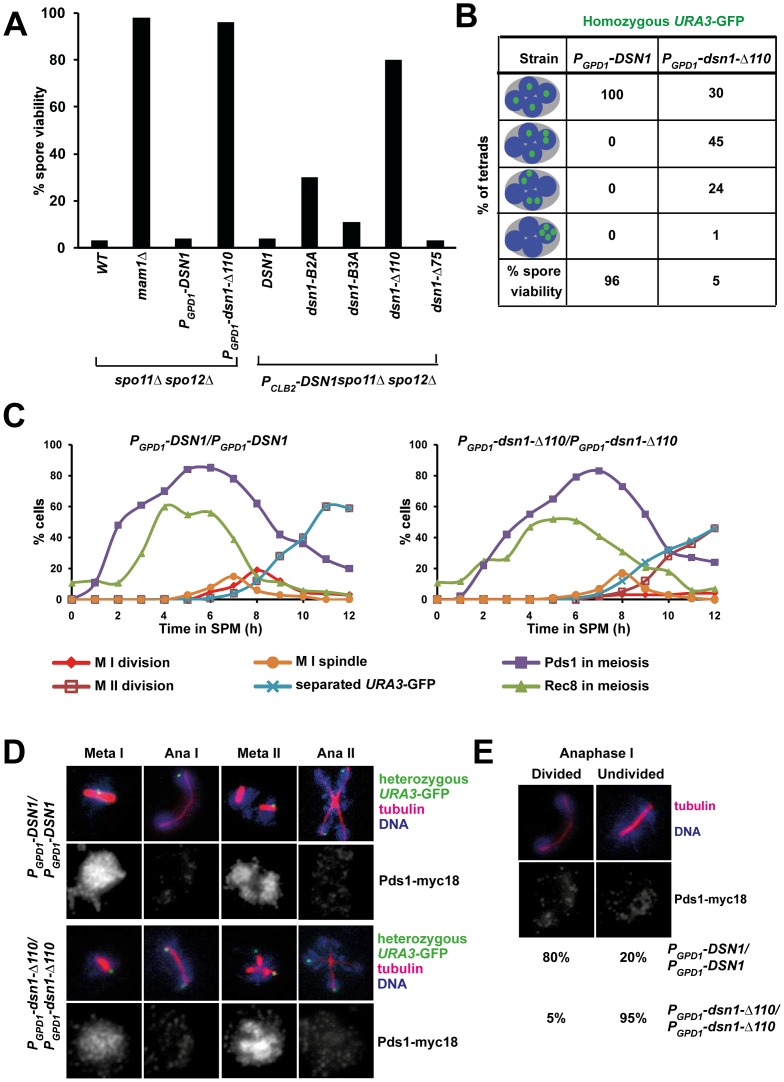
The Csm1-Interaction Domain of Dsn1 is required for the first meiotic nuclear division. A) Dyads produced by *spo11Δ spo12Δ*, *mam1Δ spo11Δ spo12Δ*, *P_GPD1_-DSN1 spo11Δ spo12Δ P_GPD1_-dsn1-Δ110 spo11Δ spo12Δ* strains and *spo11Δ spo12Δ P_CLB2_-DSN1* cells transformed with either pRS306-*DSN1* or pRS306-*DSN1-Box2A* or pRS306-*DSN1-Box3A* or pRS306-*DSN1-Δ75* or pRS306-*DSN1-Δ110* were dissected on YPD plates and grown at 30°C. Spore viability (n = 100) was scored after 3 days. B) Detection of homozygous *URA3-*GFP and DNA in tetrads produced by *P_GPD1_-DSN1 and P_GPD1_-dsn1-Δ110 cells*. Tetrads produced by the strains were dissected onto YPD plates and grown at 30°C. Spore viability (n = 100) was scored after 3 days. C–E) Immunofluorescence analysis. *P_GPD1_-DSN1* and *P_GPD1_-dsn1-Δ110* cells containing Pds1-myc18, Rec8-ha3 and heterozygous *URA3*-GFP were fixed and analysed by fluorescence microscopy. C) Nuclear division, and the percentage of cells containing Pds1, Rec8, metaphase I spindles and separated sister *URA3-*GFP dots were scored in *P_GPD1_-DSN1* and *P_GPD1_-dsn1-Δ110* cells. D) Images showing various stages of meiosis in wild type and mutant cells following fixation and fluorescence microscopy to detect *URA3*-GFP, tubulin, DNA and Pds1-myc18. E) Percentage of anaphase I cells (Pds1 negative and one bipolar spindle) with divided or undivided nuclei. Data (panels C–E) are of a single representative experiment of at least three independent repeats.

To determine whether the Csm1-interacting domain (CID) of Dsn1 is required for monopolar attachment during meiosis I, we first tested whether mutations in CID suppress the poor spore viability phenotype of *spo11Δ spo12Δ* strains. Deletion of *SPO12* causes a failure to exit from meiosis I resulting in production of dyads [21], [22]. Strains lacking *SPO11* (whose product initiates meiotic recombination by producing double-strand breaks on DNA) segregate homologs randomly [23]. However sister centromeres are co-oriented in *spo11Δ* cells and migrate towards the same spindle pole. Therefore *spo11Δ spo12Δ* strains produce dyads which have low (<5%) spore viability. Crucially disrupting mono-orientation, either by deleting or inactivating monopolin genes, rescues the spore viability of *spo11Δ spo12Δ* strains (Figure 3A) [4], [5]. We find that the poor spore viability of *P_CLB2_-DSN1 spo11Δ spo12Δ* cells is rescued by transformation with *DSN1* mutants lacking Box 2 (30%), Box 3 (11%) or the entire CID (80%), but not by transformation of either wild type *DSN1* or a *DSN1* mutant lacking the N-terminal 75 amino-acids (Figure 3A). These results suggest that the CID is required for mono-orientation of sister kinetochores during meiosis.

### The CID is required for accurate chromosome segregation during meiosis but is dispensable for mitotic growth

We then tested whether the CID is required for accurate chromosome segregation during wild type meiosis. Since expression of *dsn1-Δ110* has a dominant effect on meiotic chromosome segregation (see below) and thereby precluded us from generating strains by crossing, we constructed *dsn1-Δ110* strains by direct transformation. We expressed either full length Dsn1 or Dsn1-Δ110 (that lacks the CID) from the *GPD1* promoter that is active during both mitotic and meiotic cell cycles. *P_GPD1_-DSN1* and *P_GPD1_-dsn1-Δ110* strains were indistinguishable from *DSN1* strains in terms of benomyl sensitivity (Figure S2A) and mitotic chromosome loss rates (Figure S2B). These results indicate that neither *GPD1* promoter replacement of *DSN1* nor deletion of the CID affects kinetochore function during mitosis. This is consistent with recent biochemical studies which show that deletion of the first 172 residues of Dsn1 is dispensable for MIND complex assembly [24]. While *P_GPD1_-DSN1* and *P_GPD1_-dsn1-Δ110* strains were very similar in terms of their mitotic growth, their meiotic phenotypes were quite different. Only *P_GPD1_-dsn1-Δ110* but not *P_GPD1_-DSN1* completely rescued the poor spore viability of *spo11Δ spo12Δ* strains (Figure 3A). Whilst *P_GPD1_-DSN1* cells display spore viability (96%) comparable to wild type cells, the spore viability of *P_GPD1_-dsn1-Δ110* strains was dramatically reduced (5%) (Figure 3B). To visualize chromosome segregation, we tagged homologous chromosomes with GFP and followed their segregation by fluorescence microscopy. While 100% of nuclei in *P_GPD1_-DSN1* cells had 1 GFP dot per nucleus, 70% of *P_GPD1_-dsn1-Δ110* cells had nuclei containing more than 1 GFP dot (Figure 3B). These results indicate that the CID is dispensable for mitotic cell proliferation, but required for accurate chromosome segregation during meiosis. Notably, this is the first mutant allele of a core essential kinetochore protein that is differentially required for mitotic and meiotic divisions in any organism.

### The CID is required for the first meiotic nuclear division

To determine the precise role of the CID in meiotic chromosome segregation, we transferred *P_GPD1_-DSN1* and *P_GPD1_-dsn1-Δ110* cells to SPM. To visualize chromosome segregation, we tagged the *URA3* locus (located 30 kb from the centromere) in one of the two parental chromosome V's with GFP using the tetO/tetR system [25]. We also attached 18 copies of Myc epitope to Pds1 (securin) and 3 copies of HA epitope to Rec8 (cohesin subunit). Accumulation of Pds1 and Rec8, their destruction, and the formation of metaphase I spindles was indistinguishable in *P_GPD1_-DSN1* and *P_GPD1_-dsn1-Δ110* strains (Figure 3C). In *P_GPD1_-DSN1* cells, the progression from metaphase I-anaphase I coincided with destruction of Pds1, spindle elongation, nuclear division and co-segregation of sister *URA3* sequences towards the same spindle pole (Figure 3D). Pds1 re-accumulated in these bi-nucleate cells accompanied by formation of two sets of bipolar spindles. The progression from metaphase II-anaphase II was accompanied by a second round of Pds1 destruction, segregation of sister *URA3* sequences towards opposite poles and formation of 4 nuclei (Figure 3D). Although *P_GPD1_-dsn1-Δ110* cells formed metaphase I spindles with paired sister *URA3*-GFP signals, they failed to undergo the first nuclear division following destruction of Pds1 (Figure 3D). We observed a high proportion of mono-nucleate cells (95% compared to 20% in wild type cells) lacking Pds1 with stretched DNA and anaphase I-like spindles (Figure 3D and E). In these cells sister *URA3* sequences were frequently split (18%), suggesting that cells had attempted to bi-orient sister centromeres on the meiosis I spindle (Figure 3D). Additionally, *P_GPD1_- dsn1-Δ110* cells formed two sets of bipolar spindles in mono-nucleate cells and underwent a highly abnormal nuclear division where a single DNA mass was split along two sets of bipolar spindles resulting in four unequal DNA masses (Figure 3D). The phenotype of *P_GPD1_-dsn1-Δ110* cells is highly reminiscent to monopolin mutant cells in which sister centromeres bi-orient on the meiosis I spindle. To confirm this, we analysed meiotic chromosome segregation in *mam1Δ* and *dsn1-Δ110* strains in parallel and found that they were indeed strikingly similar (Figure S3).

### Deprotection of centromeric cohesion rescues the nuclear division defect of *dsn1-Δ110* cells

**Figure 4:**
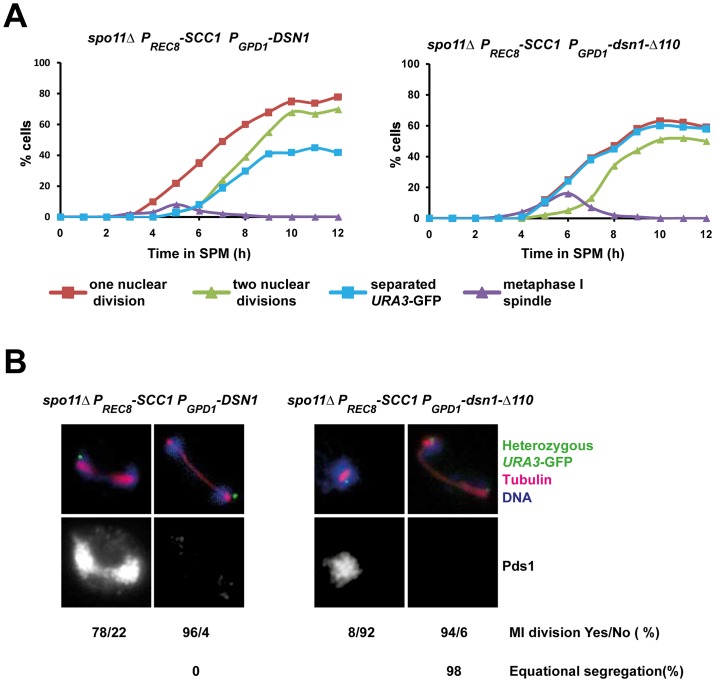
Replacement of cohesin subunit Rec8 by Scc1 rescues the nuclear division defect of *P_GPD1_-dsn1-Δ110* cells. A) Immunofluorescence analysis of meiosis in *spo11Δ P_GPD1_-DSN1 P_REC8_-SCC1* and *spo11 Δ P_GPD1_-dsn1-Δ110 P_REC8_*-*SCC1* cells containing Pds1-myc18, and heterozygous *URA3*-GFP. The percentage of cells that have undergone at least one nuclear division (red squares), a second division (green triangles), that contain a short bipolar spindle (purple triangles) and that have separated sister *URA3* sequences (blue squares) were scored. B) Nuclear division in Pds1 positive and negative cells containing a single bipolar spindle was quantified and the percentage of cells undergoing equational segregation was determined. Data shown above are of a single representative experiment of at least three independent repeats.

Monopolin mutant cells fail to undergo the first meiotic division as the microtubule pulling forces exerted on sister centromeres are resisted by centromeric cohesion which is protected during meiosis I [3]–[5]. We reasoned that if centromeric cohesion was responsible for preventing the first nuclear division in *P_GPD1_-dsn1-Δ110* cells, then its ectopic destruction should rescue the nuclear division defect in *P_GPD1_-dsn1-Δ110* cells. To test this we replaced the meiotic cohesin subunit Rec8 by Scc1 which compromises protection of centromeric cohesion, but does not affect monopolar attachment [3]. Since Scc1 cannot substitute for Rec8 in meiotic recombination, we performed our experiments in the absence of Spo11 to prevent the formation of double strand breaks. We transferred diploid *spo11Δ rec8Δ:P_REC8-_SCC1* cells that were heterozygous for *URA3*-GFP and expressing either *DSN1* or *dsn1-Δ110* into SPM. In the absence of recombination, homologs are not connected and therefore segregate randomly. However sister centromeres are mono-oriented and move towards the same spindle pole. Consistent with this anaphase I spindles were formed in the absence of Pds1 destruction and sister *URA3* sequences moved towards the same spindle pole in *spo11Δ rec8Δ:P_REC8-_SCC1* cells expressing *DSN1* (Figure 4A and 4B). In contrast, nuclei divided only after Pds1 degradation and sister *URA3* sequences moved towards opposite spindle poles in *spo11Δ rec8Δ:P_REC8-_SCC1* cells expressing *dsn1-Δ110* (Figure 4A and 4B). Thus the meiotic nuclear division defect of *dsn1-Δ110* cells can be rescued by deprotection of centromeric cohesion. These results formally demonstrate that the CID is required for monopolar attachment of sister kinetochores during meiosis I.

### The CID is required for association of monopolin with kinetochores

**Figure 5:**
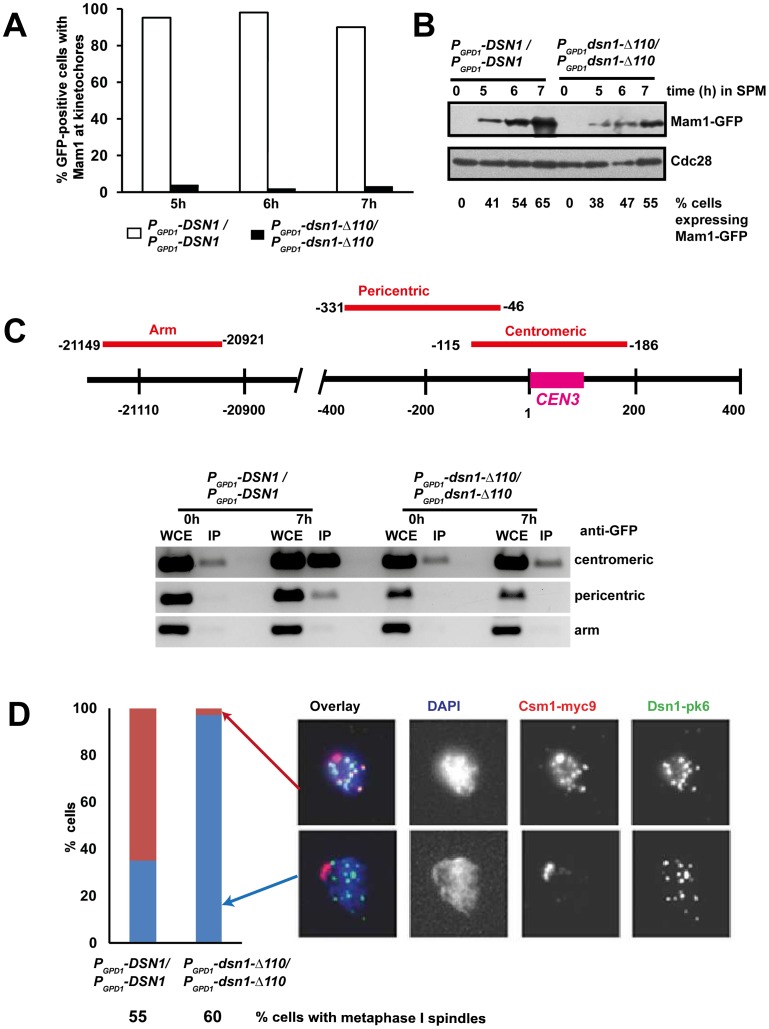
The Csm1-Interaction Domain of Dsn1 is required for monopolin association with kinetochores. *MAM1-GFP MTW1-RFP P_CLB2_-CDC20* cells expressing either *DSN1* or *dsn1-Δ110* were induced to enter meiosis. Mam1 association with kinetochores was measured using GFP fixation assay and Chromatin Immunoprecipitation (ChIP). A) Percentage of cells showing co-localization of Mam1-GFP signals with Mtw1-RFP signals is shown (n = 100). B) Protein extracts from the cultures was analysed by Western blotting using anti-GFP and anti-Cdc28 antibody as a loading control. C) ChIP analysis of DNA in whole cell extracts (WCE) or following immunoprecipitation of Mam1 with anti-GFP antibodies (IP) from extracts of *P_GPD1_-DSN1* and *P_GPD1_-dsn1-Δ110* strains after zero and 7 hours incubation in SPM. PCR was performed using primers specific for centromeric, pericentric and arm sequences of chromosome III indicated in the schematic. D) *CSM1-myc9 P_CLB2_-CDC20* cells expressing either Dsn1-pk6 or Dsn1-Δ110-pk6 were induced to enter meiosis. After 7 hours incubation in SPM, association of Csm1 with kinetochores was assayed by chromosome spreading using anti-PK and anti-myc antibodies. DNA was visualized using DAPI. Percentage of cells with metaphase I spindle after 7 h in SPM was measured by immunofluorescence and is indicated below the strain's genotype. Data of a single representative experiment of at least three independent repeats are presented above.

To test whether the CID is required for association of monopolin with kinetochores during meiosis I, we induced *MAM1-GFP MTW1-RFP P_CLB2_-CDC20* diploid cells expressing either wild type *DSN1* or *dsn1-Δ110* to enter meiosis and examined the binding of Mam1 to kinetochores by fluorescence microscopy and chromatin immunoprecipitation (ChIP). Mam1 was expressed after 5 hours following transfer to SPM in both *P_GPD1_*-*DSN1* and *P_GPD1_-dsn1-Δ110* strains (Figure 5B). While Mam1-GFP dots coincided with Mtw1-RFP signals in 90% of *P_GPD1_*-*DSN1* cells, co-localisation of Mam1 and Mtw1 was observed in only 3% of *P_GPD1_-dsn1-Δ110* cells (Figure 5A). To rule out the possibility that lack of Mam1 binding to kinetochores in the *dsn1-Δ110* strain was due to our fixation protocol, we also performed time-lapse video microscopy of wild type and *dsn1-Δ110* cells after 6 h in SPM. In 100% of wild type cells (n = 60) analysed, Mam1-GFP signal was strongly enriched at kinetochore and this enrichment was maintained for the entire length of imaging analysis (Figure S4 and Supplemental Video S1). In contrast in 100% of *dsn1-Δ110* cells (n = 200) the Mam1-GFP signals were diffuse and did not show any enrichment at kinetochores during the entire experiment (Figure S4 and Supplemental Video S2).

To confirm that CID is required for monopolin binding to kinetochores, we performed Chromatin Immunoprecipitation (ChIP). Consistently, we find that Mam1 binds to centromeric and pericentric DNA in *P_GPD1_*-*DSN1*, but not in *P_GPD1_-dsn1-Δ110*, cells by ChIP (Figure 5C). We also examined the association of Csm1 with kinetochores in *P_GPD1_*-*DSN1* and *P_GPD1_-dsn1-Δ110* cells by chromosome spreading. Diploid *CSM1-myc9 P_CLB2_-CDC20* cells expressing either Dsn1-pk6 or Dsn1-Δ110-pk6 were induced to enter meiosis by transferring them to SPM for 7 hours. Chromosome spreads were prepared and stained with anti-myc and anti-PK antibodies to detect Csm1 and Dsn1, respectively. We find that Csm1 foci coincide with Dsn1 in 70% of wild type cells, but in only 2% of mutant cells, even though comparable proportions of wild type and mutant cells formed metaphase I spindles (Figure 5D). Together these results indicate that the CID is required for stable association of monopolin with kinetochores during meiosis I.

We also note that the CID is required for association of Csm1/Lrs4 complex with kinetochores during mitotic anaphase (Figure S5). It was recently reported that the binding of Csm1/Lrs4 complex to kinetochores during anaphase is required for accurate chromosome segregation during mitosis [26]. However this notion is inconsistent with our observation that *P_GPD1_-dsn1-Δ110* cells do not have an increased chromosome loss rate (Figure S2B). However, we find that, consistent with an earlier report [4], *lrs4Δ* strain was indistinguishable from wild type strain in terms of its chromosome loss rate (Figure S2B).

We observed that *dsn1-Δ110* had a semi-dominant effect on meiotic chromosome segregation and spore viability (Figure S6A and S6B). The semi-dominant effect of *dsn1-Δ110* was not due to sub-optimal association of monopolin with kinetochores (Figure S6C) but is consistent with the notion that Dsn1-Δ110 protein binds efficiently to kinetochores, but interferes with the cross-linking of sister kinetochores (Figure S6D).

## Discussion

By performing a microscopic screen, we have identified the MIND complex to be required for monopolin association with kinetochores. In particular, we have demonstrated an interaction between monopolin subunit Csm1 and the N-terminal domain of Dsn1. Furthermore we have shown the CID (the N-terminal 110 amino acid residues of Dsn1) is required for mono-orientation of sister kinetochores and localization of monopolin to kinetochores.

**Figure 6:**
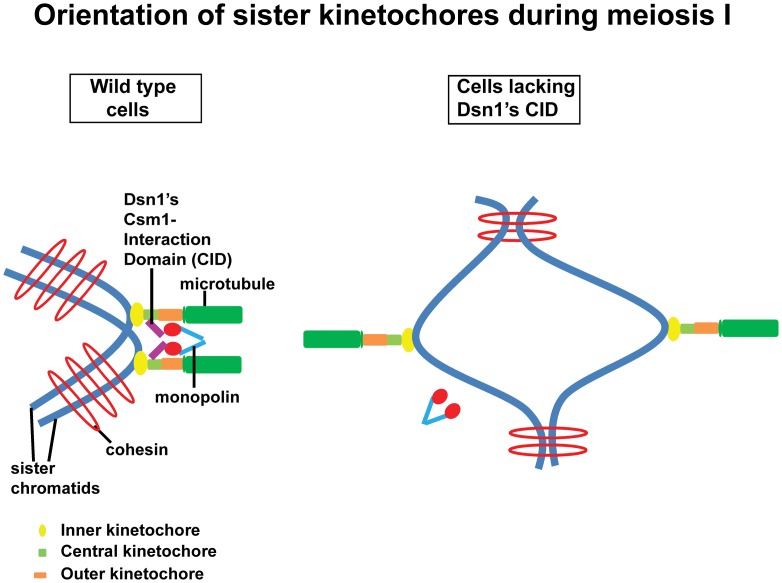
Role of the CID in monopolar attachment of sister kinetochores during meiosis I. Kinetochores are composed of three layers namely inner (yellow oval), central (green rectangle) and outer (orange rectangle). They are assembled at centromeres on sister chromatids (in blue). The outer kinetochore interacts with the growing ends of microtubules (in green). In wild type cells, monopolar attachment is achieved by interaction of the Csm1-Interaction Domain (purple) in Dsn1 (a subunit of the central kinetochore MIND complex) with the globular domain of Csm1 (red), a monopolin (V-shaped structure in blue) subunit. In cells expressing a form of Dsn1 lacking the Csm1-Interaction Domain, monopolins fail to bind to kinetochores and consequently the sister kinetochores are bi-oriented.

Cross-linking of kinetochores could be achieved by association of monopolin with the CID of Dsn1. Alternatively, the CID of Dsn1 could simply target monopolins to kinetochore which then crosslink kinetochores *via* a different mechanism. The fact that we see a dominant effect of Dsn1 lacking CID on meiotic chromosome segregation is consistent with the former possibility. Based on the structural data of the monopolin complex [9] and our findings, we propose that monopolin crosslinks sister kinetochores during meiosis I in wild type cells and this is mediated by an interaction between Csm1's globular domain and the CID in Dsn1(Figure 6). Sister kinetochores are thereby constrained to face the same spindle pole and thus mono-oriented on the meiosis I spindle. In cells lacking the CID, monopolin fails to associate with kinetochores and the sister kinetochores are consequently bi-oriented on the meiosis I spindle (Figure 6).

Quantitative imaging microscopy has revealed that a single budding yeast kinetochore has 6–8 copies of the MIND complex [27]. If monopolin works by cross-linking Dsn1 molecules, it is intriguing how monopolin distinguishes Dsn1 molecules on the same kinetochore from those bound to two different kinetochores. The catalytic activity of casein kinase Hrr25 is required for monopolar attachment but not for kinetochore recruitment [5]. It will be interesting to determine whether MIND complex is phosphorylated by Hrr25 kinase during meiosis I. Hyperphosphorylation of Lrs4 by the Dbf4-dependent kinase Cdc7 facilitates monopolin binding to kinetochores [8]. It will be worth investigating whether Lrs4 hyperphosphorylation enhances the Csm1-CID interaction.

Intriguingly, the CID is not present in fission yeast Dsn1 (Mis13) even though fission yeast expresses orthologues of Csm1 (Pcs1) and Lrs4 (Mde4). Notably Pcs1 interacts with Cnp3 (homolog of Mif2/CENP-C) and requires Cnp3 for its stable association with kinetochores [28]. However, the domain of Pcs1 that interacts with Cnp3 is not present in budding yeast Mif2 (Figure S7). Moreover, we and others have not detected any interaction between Mif2 and Csm1 *in vitro* ([10] and data not shown). It is quite possible that budding and fission yeast monopolin complexes have distinct kinetochore binding sites. This is not unprecedented as there are many species–specific variations in the kinetochore architecture despite a conserved building scheme. For instance, the vertebrate Ndc80 complex contacts the Mis12 complex by interacting with the C-terminal domain of Nsl1 subunit [29]. However the budding and fission yeast Nsl1 homologs do not have this interaction domain.

It has been suggested that Pcs1/Mde4 establishes chromosome bi-orientation in fission yeast by targeting condensin to the centromere [13]. Curiously, the Csm1/Lrs4 complex targets condensin to rDNA regions in budding yeast [30]. Unravelling the precise relationship between the abilities of monopolins to mono-orient kinetochores and direct condensin to defined regions of the chromosome is a crucial challenge for the future. Notably, monopolar attachment of sister kinetochores in plants occurs by cross-linking of Mis12 complexes [31]. It will be important to determine whether a mechanism involving cross-linking of Mis12 complexes also operates during co-orientation of sister kinetochores during mammalian meiosis.

## Materials and Methods

### Cell culture

Diploid SK1 strains were used for most experiments except for the 2-hybrid assays (AH109 strain -Clontech) and the chromosome loss assay experiments (BY4741). A list of strains used is provided in the Table S1. PCR-based modifications of the yeast genome including gene deletions, epitope tagging and promoter replacements were performed using standard yeast techniques. Meiotic cultures were performed at 30°C as previously described [32].

### Fixation and fluorescence microscopy

One ml sporulation culture was spun in a microfuge tube at 13,000 rpm for a minute and the cells were re-suspended in 100 µl fixative (4% paraformadehyde, 3.4% sucrose) and incubated at room temperature for 15 minutes. Cells were then spun and washed once in Solution I (100 mM Potassium phosphate pH 7.5 1.2 M Sorbitol) and then resuspended in 100 µl of Solution I. Cells were sonicated for 5 sec and then 3 µl cells were mounted on a slide for fluorescence microscopy. Images were acquired using an inverted microscope (TE-2000 Nikon) with a 100×1.49 NA objective lens equipped with a cooled charge-coupled device camera (CoolSNAP HQ2; Photometrics). 16 z-stack (spacing = 0.2 µm) were used at exposure times of 1 sec for Cy3, Cy5, RFP and Alexa Fluor 488/GFP and 0.25 sec for DAPI. Images were analyzed using MetaMorph (version 7.5.2.0; MAG Biosystems Software).

### Time-lapse video microscopy

Cells were induced to enter meiosis by transferring them to sporulation medium for 6 hours. Then 20 µl of cells were added onto a Y04D CellASIC plate (CellASIC ONIX microfluidic perfusion system) and imaged inside an environmental chamber set at 30°C. A flow rate of 8 psi was used to load the cells and a steady-state flow rate of 2 psi was used for the duration of the experiment. Time-lapse microscopy was carried out using a Personal DeltaVision (Applied Precision) with solid-state illumination, using associated proprietary software (SoftWoRx software; version 4.0.0, Applied Precision). Images were captured using an UPLS Apochromat 1.4 numerical aperture, ×100 magnification oil immersion objective (Olympus), standard DeltaVision filter sets FITC (Excitation 490 nm, Emission 525 nm) and TRITC (Excitation 490 nm, Emission 525 nm) yielding approximate resolutions (Rayleigh's d) of ∼229 nm and 264 nm in the xy, respectively, whereas axial resolutions were approximately 811 and 935 nm. Photon detection was carried out using a Cascade2 1 K EMCCD camera (Photometrics) using a gain of 4.00 and no binning. Effective pixel size was ∼0.0645 µm in the xy. Images were taken using exposure times of 0.1 sec and 32% transmission (FITC) and 0.15 sec exposure (TRITC). Final images for sporulation were carried out with DIC, 32% transmission and 0.08 sec exposure. 8 z-stacks of 0.5 µm thickness were taken of each nucleus. Images were recorded every 10 minutes for 2 hours.

### Chromatin Immunoprecipitation (ChIP)

Five ml of 37% formaldehyde was added to 50 ml yeast culture and the mixture was incubated at 30°C for 30 minutes. 2.5 ml of 2.5 M glycine was added and the mixture was incubated further for 5 minutes. Cells were spun at room temperature at 3,000 rpm (1,620 *g*) for 3 minutes. The supernatant was discarded and cells were resuspended in 25 ml 1× PBS and pelleted at 3,000 rpm (1620 *g*) for 3 minutes. The supernatant was discarded and cells were resuspended in 1 ml 1× PBS and transferred to a fresh eppendorf tube. Cells were spun at room temperature at 10,000 rpm (9,500 *g*) for 2 minutes and the pellet was frozen at −80°C. Cells were re-suspended in lysis buffer A (50 mM HEPES-KOH pH 7.5, 140 mM NaCl, 1 mM EDTA, 1% Triton X-100, 0.1% sodium deoxycholate, 1 mM PMSF, EDTA free complete mini protease inhibitor mix). An equal volume of glass beads was added to the cells and the mixture was vortexed in a bead beater (1 minute on and 1 minute off, 20 times). This was followed by sonication (30 sec on and 30 sec off, 6 times). The lysate was cleared using protein G-Sepharose beads for 1 hour at 4°C. The cleared lysate was incubated with mouse anti-GFP antibody overnight at 4°C followed by 2 hours incubation with protein G-Sepharose beads on a rotary wheel at 4°C. The beads were then washed 3 times with lysis buffer A. Proteins were eluted off the beads by heating the beads in TES buffer (50 mM Tris pH 8.0, 1 mM EDTA, 10% SDS) overnight at 65°C. The beads were discarded and the supernatant was treated with RNAse A for 1 hour at room temperature and proteinase K in 50 mM Tris pH 8.0 for 2 hours at 37°C. DNA was purified using Qiagen columns as per the manufacturer's instructions. Primers used for amplifying the centromeric, pericentric and arm regions of Chromosome III have been previously described [4]. Whole-cell extract (WCE) DNA was diluted 25 times and 1 µl was used as a template in a 50 µl Polymerase Chain Reaction (PCR). The immunoprecipitated DNA was diluted 5 times and 1 µl was used in a 50 µl PCR. The PCR products were run on a 2% agarose gel and visualized by staining with ethidium bromide.

### In vitro interaction assays

Either pMAL-c2x vector or pMAL-c2x vector containing Csm1 ORF (and its variants) was introduced into *E. coli* Rosetta (DE3)pLysS Competent Cells (Novagen) by transformation. Transformants were grown in 100 ml LB medium at 37°C until the OD_600_ was 0.6. Expression of Csm1-MBP/MBP was induced by addition of IPTG (0.2 mM) and cultures were incubated for 3 hours at 37°C. Cells were harvested and resuspended in 5 ml lysis buffer B (50 mM HEPES-KOH pH 7.5, 140 mM NaCl, 1 mM PMSF, Roche EDTA-free complete mini protease inhibitor mix) and sonicated for 4 minutes (1 min on, 1 min off) using the Sonics. Cell lysates were spun at 18,000 rpm for 30 minutes. The supernatant was collected and mixed with 500 µl of Amylose resin (New England Biolabs) and incubated for 1 hour at 4°C. Beads were pelleted and washed with lysis buffer B three times and then resuspended in 500 µl of lysis buffer B. This was used for the binding assay (see below). Dsn1 or its truncated version Dsn1 (111–576), carrying 6 copies of PK tag attached to its C-terminus was expressed in *S. cerevisiae* from the *GPD1* promoter. Yeast cells from an overnight culture were inoculated into 50 ml YEPD with a starting OD of 0.2 and incubated at 30°C in a shaker at 170 rpm. Cells were harvested and resuspended in 600 µl of lysis buffer A and were mixed with an equal volume of glass beads. This mixture was vortexed using the VIBRAX-IKA for 16 minutes (1 min on, 1 min off, 4 times, 4 cycles). The lysate was separated from the cell debris by spinning at 14,000 rpm for 10 minutes. 100 µl of lysate was mixed with 100 µl of Csm1-MBP/MBP beads (purified above) and kept at 4°C for 30 minutes. Beads were then washed thrice with 1 ml of lysis buffer A containing 1% NP40. Beads were re-suspended in 100 µl of 2xSDS-sample buffer and incubated at 95°C for 5 minutes to elute the bound proteins.

### Yeast 2-hybrid analysis

The ORF encoding *CSM1* was cloned into EcoRI and SalI sites of pGADT7 (Clontech). The ORF encoding Dsn1 was cloned into NcoI and BamHI sites of pGBKT7 (Clontech). Mutant versions of Dsn1 in pGBKT7 were generated by gap-repair. Briefly oligos with 50 base homology to regions upstream and downstream of BamHI and NcoI sites respectively of pGBKT7 at their 5′ ends followed by 15 nucleotides specific to *DSN1* were designed to amplify specific regions of *DSN1* ORF. 1 µg of the PCR product was co-transformed with 100 ng of BamHI/NcoI digested pGBKT7 into AH109 cells containing either pGADT7 or pGADT7-*CSM1*. Transformants were tested for interaction by replica plating them on SD–LEU-TRP-ADE-HIS plates. Genomic DNA from four randomly chosen transformants was used as a template for amplifying the insert and the PCR products obtained were sequenced to confirm the identity of the mutants created by gap repair. Details of oligonucleotides used in the study are available upon request.

### Immunoblotting, chromosome spreads and *in situ* immunofluorescence

Immunoblotting, chromosome spreads and *in situ* immunofluorescence were performed as previously described [32]. For Westerns, mouse anti-PK antibody (Serotec), Goat anti-Cdc28 antibody (Santa Cruz) and mouse anti-GFP antibody (Roche) were all used at 1∶1000 dilutions. For staining *in situs*, mouse anti-myc 9E10 (1∶500), mouse anti-HA16B12 (1∶500) and rat anti-tubulin YOL1/34 (1∶500) were used. For chromosome spreads the rabbit anti-myc (Gramsch; 1∶500) and mouse anti-PK (1∶500) antibodies were used.

### Chromosome loss assay

Mitotic chromosome loss assay was performed as previously described [33]. Strains used for chromosome loss assay harbour the *ade2-101* ochre mutation and a supernumerary chromosome that contains *URA3* marker and *SUP11* (an ochre-suppressing tRNA). Cells containing the supernumerary chromosome produce white colonies as *SUP11* suppresses the *ade2-101* ochre allele. Loss of the supernumerary chromosome results in the formation of a red sector in an otherwise white colony. For the assay, cells were grown overnight on minimal medium lacking uracil and then plated at a density of 300 colonies per plate on YEP (1.1%Yeast extract, 2.2% Peptone and 2% Glucose) medium. After 2–3 days of incubation at 30°C, colonies that were at least half red were counted. The chromosome loss rate was computed by dividing the number of half-sectored colonies by the total number of colonies scored.

## Supporting Information

Figure S1 The Spc24 and Ask1 proteins are efficiently depleted in *P_CLB2_-ha_3-_SPC24* and *P_CLB2_- ha_3_-ASK1* strains respectively during meiosis. Western blots of protein extracts from mitotic/meiotic cultures of wild type, *P_CLB2_-ha_3_-SPC24* and *P_CLB2_-ha_3_-ASK1* strains were probed using anti-HA and anti-Cdc28 antibodies (loading control).
(PDF)

Figure S2 The Csm1-Interaction Domain of Dsn1 is not required for accurate chromosome segregation during mitosis. A) Log-phase cultures of wild type, *P_GPD1_-DSN1*, *P_GPD1_-dsn1-Δ110* and *ctf19Δ* strains were serially diluted 10-fold and spotted on YEPD plates with or without benomyl (15 µg/ml). Growth was monitored after incubation at 30°C for 3 days. Please note that *ctf19Δ* cells are slightly sensitive to benomyl compared to wild type cells whereas the *P_GPD1_-dsn1-Δ110* cells are as resistant to benomyl as *P_GPD1_-DSN1* cells. B) Overnight cultures of wild type, *P_GPD1_-DSN1*, *P_GPD1_-dsn1-Δ110*, *lrs4Δ* and *rts1Δ* strains carrying the *SUP11*-marked supernumerary chromosome and *ade2-101* ochre allele were grown in –URA medium in triplicates and then plated for single colonies on YEP plates (at a cell density of 300 colonies per plate). Plates were incubated at 30°C for 3 days and the fraction of colonies (N = 5000) that showed at least half sectoring was calculated. While *rts1*Δ strains (used as a control) had a 200-fold increase in chromosome loss rate, the *P_GPD1_-dsn1-Δ110* and *lrs4Δ* strains were indistinguishable from their wild type controls.
(PDF)

Figure S3 The meiotic chromosome segregation phenotypes of *P_GPD1_-dsn1-Δ110* and *mam1Δ* cells are strikingly similar. Wild type, *mam1Δ*, *P_GPD1_-DSN1* and *P_GPD1_-dsn1-Δ110* cells containing Pds1-myc18, and heterozygous *URA3*-GFP were induced to enter meiosis by transferring them to SPM. A) Hourly samples of the four strains were fixed subjected to immunostaining and analysed by fluorescence microscopy. Nuclear division and the percentage of cells containing Pds1 and separated sister *URA3-*GFP dots were scored in the four strains and graphically presented. B) Percentage of Pds1 negative cells with split/unsplit nucleus for the four strains was determined and graphically presented.
(PDF)

Figure S4 Live cell imaging confirms that the CID is required for Mam1 localization to kinetochores. Stills (maximum intensity projections) from live cell imaging of Mam1-GFP and Mtw1-RFP in cells expressing Dsn1 or Dsn1-Δ110 are shown. (Videos are available at Supplemental videos S1 and S2). Mam1-GFP is in green and kinetochores (Mtw1-RFP) are in red. Cells with accumulation of Mam1-GFP and either separated kinetochores during the time lapse or had already separated kinetochores were chosen for analysis. Arrows indicate cells with enriched Mam1-GFP at kinetochores. White bar indicates a length of 5 µm.
(PDF)

Figure S5 The Csm1-Interaction Domain of Dsn1 is required for association of Csm1/Lrs4 complex during mitotic anaphase. *NDC10-ha6 LRS4-myc9 P_MET3_-CDC20* cells containing either *P_GPD1_-DSN1* or *P_GPD1_-dsn1-Δ110* were arrested in metaphase by addition of methionine to the growth medium. Cells were then released into anaphase by transferring them to a growth medium lacking methionine. A) Chromosome spreads were prepared and stained with anti-myc and anti-HA antibodies and DNA was visualized by staining with DAPI. The percentage of nuclei displaying i) >50% co-localization of Ndc10 and Lrs4 foci (green bar) ii) <50% co-localization of Ndc10 and Lrs4 foci (red bar) iii) No co-localization (blue bar) was measured. Representative images of nuclei belonging to three categories are depicted. B) Kinetics of nuclear division (scored by DAPI staining) is presented. C) Kinetics of anaphase spindle (spindle length>3 µm) assembly/disassembly and budding index are presented.
(PDF)

Figure S6 Expression of a *dsn1* mutant lacking the Csm1-Interaction Domain dominantly interferes with monopolar attachment. A) Analysis of nuclear division and separation of *URA3*-GFP sequences during meiosis in *P_GPD1_-DSN1/P_GPD1_-DSN1* and *P_GPD1_-DSN1/P_GPD1_-dsn1-Δ110* cells expressing Pds1-myc18. Note that *P_GPD1_-DSN1/P_GPD1_-dsn1-Δ110* cells fail to undergo the first meiotic nuclear division as shown by the absence of binucleates (2N) and separate *URA3* dots before the appearance of tetranucleates. B) Percentage of anaphase I cells (Pds1 negative and one bipolar spindle) with divided or undivided nuclei. Spore viabilities of the two strains were determined (n = 100) and are indicated below their genotypes. C) *MAM1-GFP MTW1-RFP P_CLB2_-CDC20* cells expressing either Dsn1 or Dsn1-Δ110 or Dsn1 and Dsn1-Δ110, were induced to enter meiosis by transferring them to SPM. Mam1 association with kinetochores was measured using GFP fixation assay after 5, 6 and 7 hours into SPM. D) An explanation why Dsn1 lacking the CID has a dominant negative effect on monopolar attachment. Kinetochores (brown) are assembled at centromeres on sister chromatids (black). Monopolin (in red) co-orients sister kinetochores in wild type cells by interacting with the CID in Dsn1, a part of the MIND complex (in blue). In cells expressing both Dsn1 and Dsn1-Δ110, monopolins fail to crosslink kinetochores as one of the two sister kinetochores lacks a monopolin-binding site (in pink).
(PDF)

Figure S7 The Pcs1-interacting domain in fission yeast Cnp3 is not conserved in budding yeast Mif2. The positions of the N-terminal domain, CENP-C homology domain (containing the CENP-C signature sequence), DNA-binding domain and dimerization domain in Mif2 and Cnp3 are indicated. Mif2 lacks a region with sequence similarity to the Pcs1-interacting domain in Cnp3.
(PDF)

Table S1 All yeast strains are derivatives of SK1 and have the following markers, unless otherwise stated. *ho::LYS2/ho::LYS2, ura3/ura3, leu2::hisG/leu2::hisG, trp1::hisG/trp1::hisG, his3::hisG/his3::hisG, lys2/lys2.* Markers are homozygous in diploid strains unless otherwise stated.
(PDF)

Video S1 Time-lapse imaging of *MAM1-GFP MTW1-RFP P_CLB2_-CDC20* cells expressing *DSN1* was performed after 6 h in SPM. Images were acquired at an interval of 10 minutes for 2 hours. Scale bar, 15 µm.
(MOV)

Video S2 Same as Video S1 except that the imaging was done with a strain expressing *dsn1-Δ110* instead of *DSN1*.
(MOV)
