# Supplementary figures and images for: Monopolin Subunit Csm1 Associates with MIND Complex to Establish Monopolar Attachment of Sister Kinetochores at Meiosis I
Source: PLoS Genet. 2013 Jul 4;9(7):e1003610. doi: 10.1371/journal.pgen.1003610 (PMC3701701; doi:10.1371/journal.pgen.1003610)

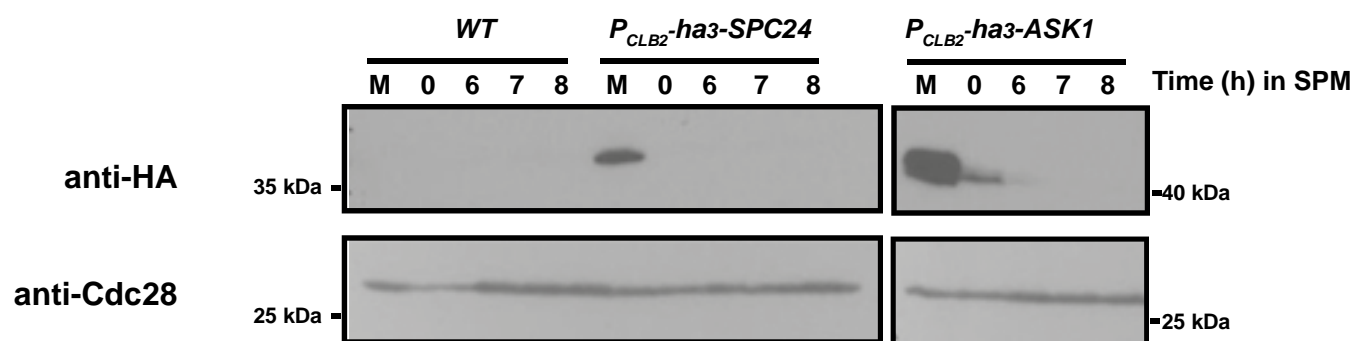

**Figure S1**

Supplement: Figure S1 — The Spc24 and Ask1 proteins are efficiently depleted in PCLB2-ha3-SPC24 and PCLB2- ha3-ASK1 strains respectively during meiosis. Western blots of protein extracts from mitotic/meiotic cultures of wild type, PCLB2-ha3-SPC24 and PCLB2-ha3-ASK1 strains were probed using anti-HA and anti-Cdc28 antibodies (loading control). (PDF) [file pgen.1003610.s001.pdf]

**A**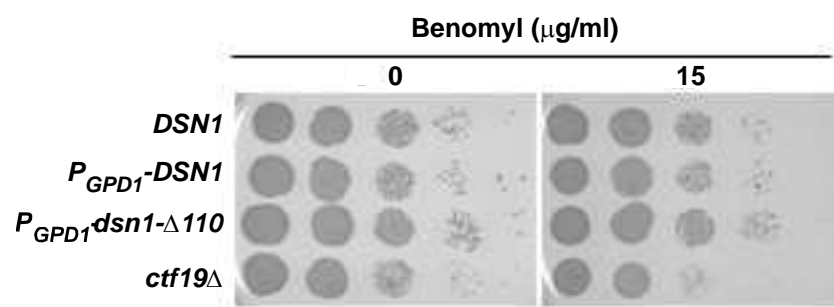**B**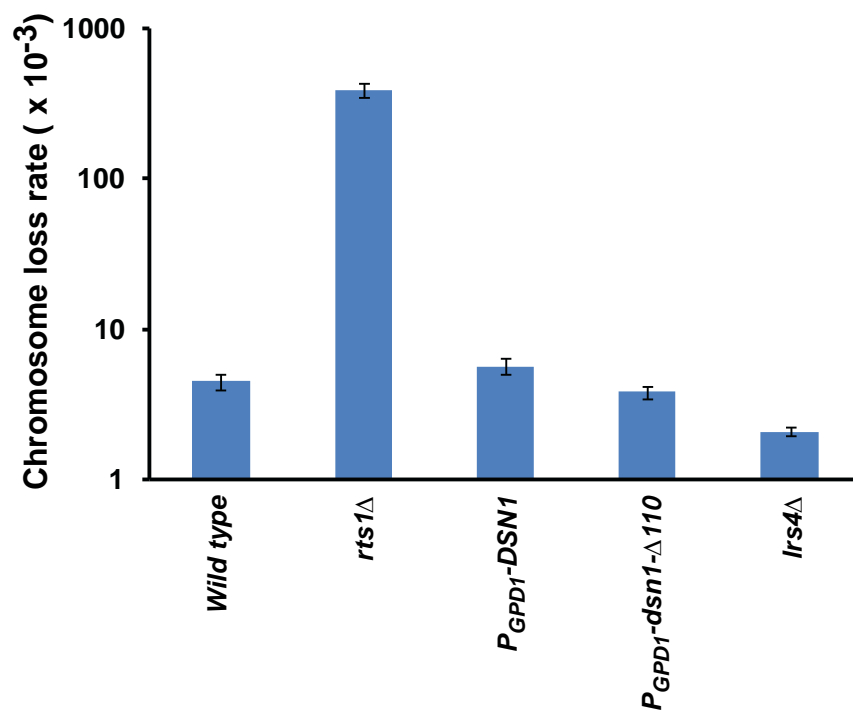**Figure S2**

Supplement: Figure S2 — The Csm1-Interaction Domain of Dsn1 is not required for accurate chromosome segregation during mitosis. A) Log-phase cultures of wild type, PGPD1-DSN1, PGPD1-dsn1-Δ110 and ctf19Δ strains were serially diluted 10-fold and spotted on YEPD plates with or without benomyl (15 µg/ml). Growth was monitored after incubation at 30°C for 3 days. Please note that ctf19Δ cells are slightly sensitive to benomyl compared to wild type cells whereas the PGPD1-dsn1-Δ110 cells are as resistant to benomyl as PGPD1-DSN1 cells. B) Overnight cultures of wild type, PGPD1-DSN1, PGPD1-dsn1-Δ110, lrs4Δ and rts1Δ strains carrying the SUP11-marked supernumerary chromosome and ade2-101 ochre allele were grown in –URA medium in triplicates and then plated for single colonies on YEP plates (at a cell density of 300 colonies per plate). Plates were incubated at 30°C for 3 days and the fraction of colonies (N = 5000) that showed at least half sectoring was calculated. While rts1Δ strains (used as a control) had a 200-fold increase in chromosome loss rate, the PGPD1-dsn1-Δ110 and lrs4Δ strains were indistinguishable from their wild type controls. (PDF) [file pgen.1003610.s002.pdf]

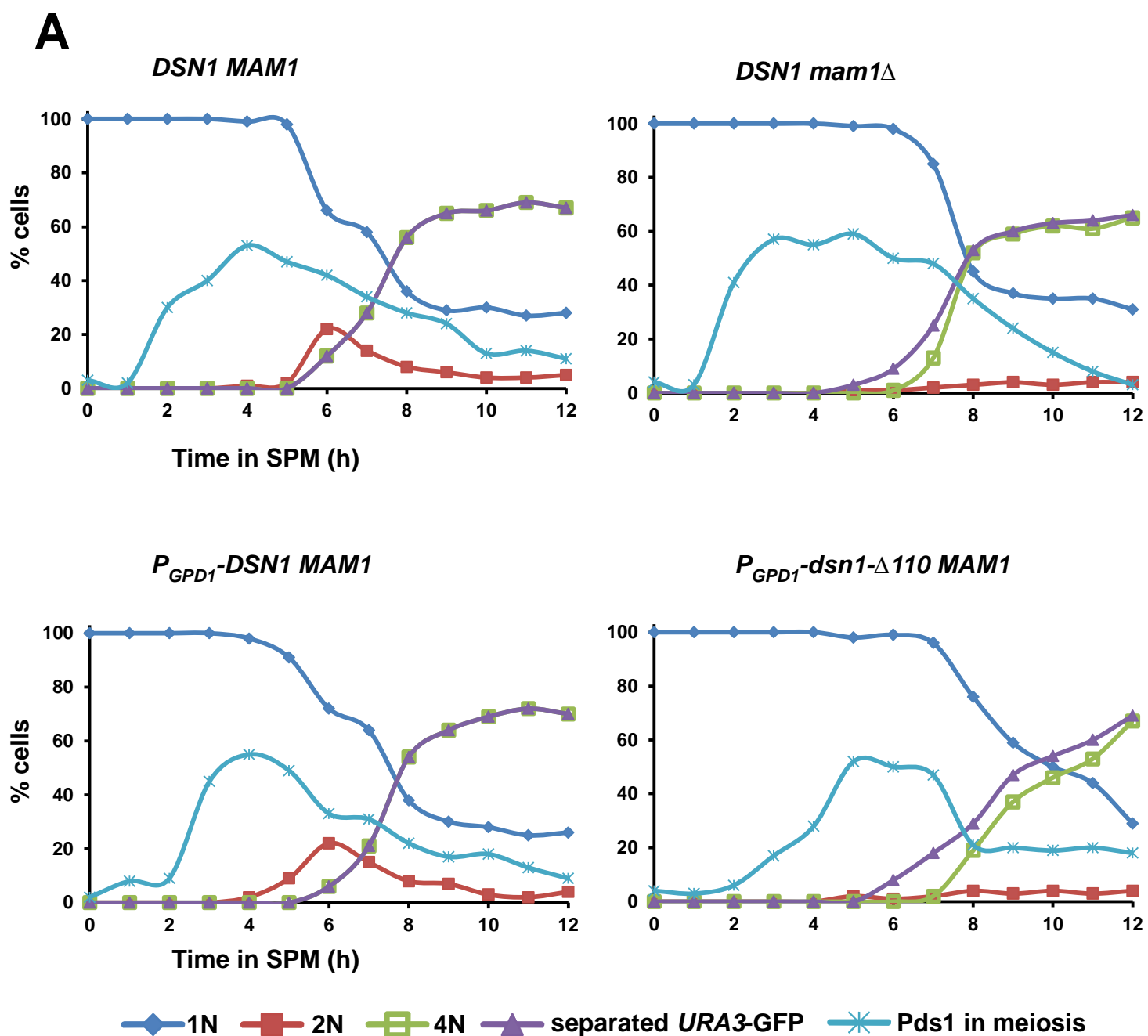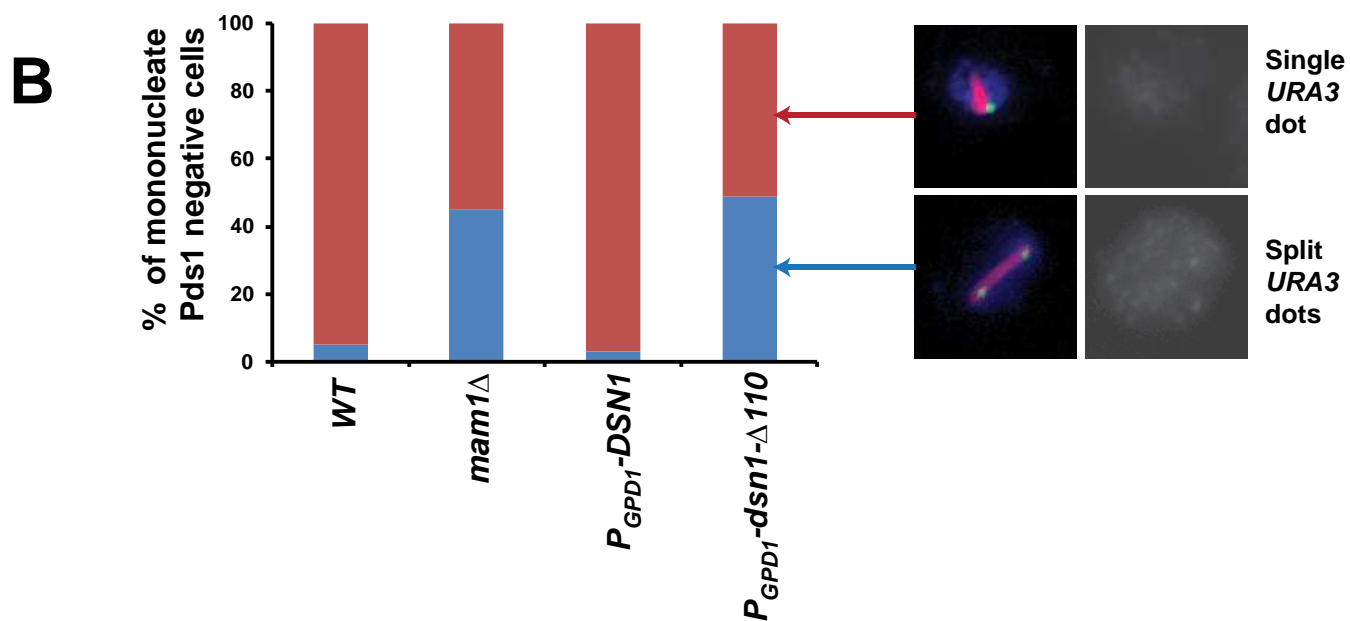

**Figure S3**

Supplement: Figure S3 — The meiotic chromosome segregation phenotypes of PGPD1-dsn1-Δ110 and mam1Δ cells are strikingly similar. Wild type, mam1Δ, PGPD1-DSN1 and PGPD1-dsn1-Δ110 cells containing Pds1-myc18, and heterozygous URA3-GFP were induced to enter meiosis by transferring them to SPM. A) Hourly samples of the four strains were fixed subjected to immunostaining and analysed by fluorescence microscopy. Nuclear division and the percentage of cells containing Pds1 and separated sister URA3-GFP dots were scored in the four strains and graphically presented. B) Percentage of Pds1 negative cells with split/unsplit nucleus for the four strains was determined and graphically presented. (PDF) [file pgen.1003610.s003.pdf]

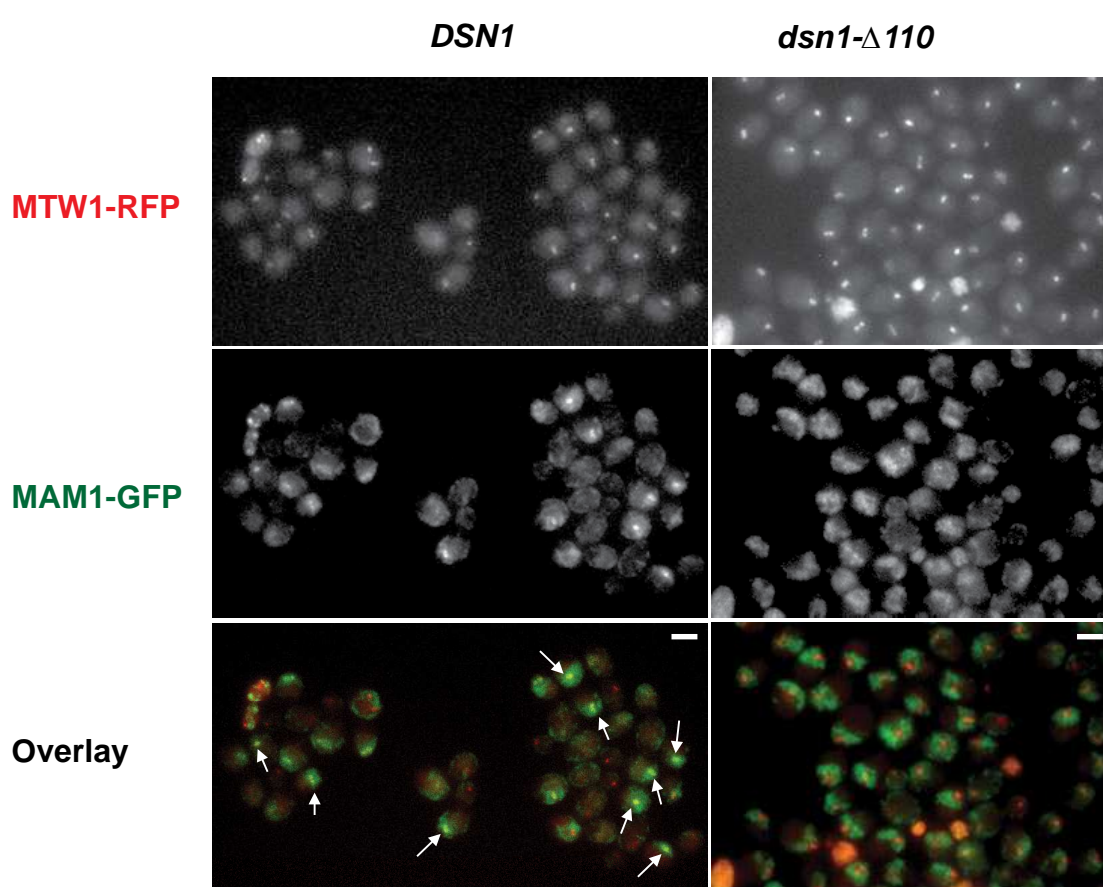

**Figure S4**

Supplement: Figure S4 — Live cell imaging confirms that the CID is required for Mam1 localization to kinetochores. Stills (maximum intensity projections) from live cell imaging of Mam1-GFP and Mtw1-RFP in cells expressing Dsn1 or Dsn1-Δ110 are shown. (Videos are available at Supplemental videos S1 and S2). Mam1-GFP is in green and kinetochores (Mtw1-RFP) are in red. Cells with accumulation of Mam1-GFP and either separated kinetochores during the time lapse or had already separated kinetochores were chosen for analysis. Arrows indicate cells with enriched Mam1-GFP at kinetochores. White bar indicates a length of 5 µm. (PDF) [file pgen.1003610.s004.pdf]

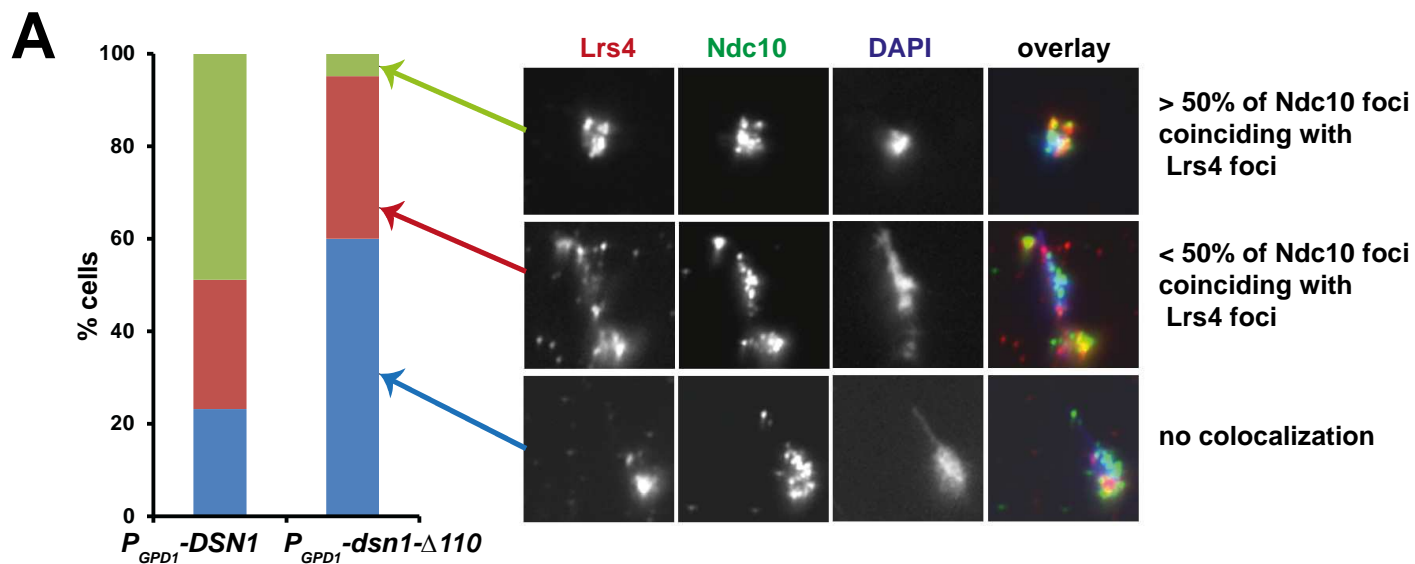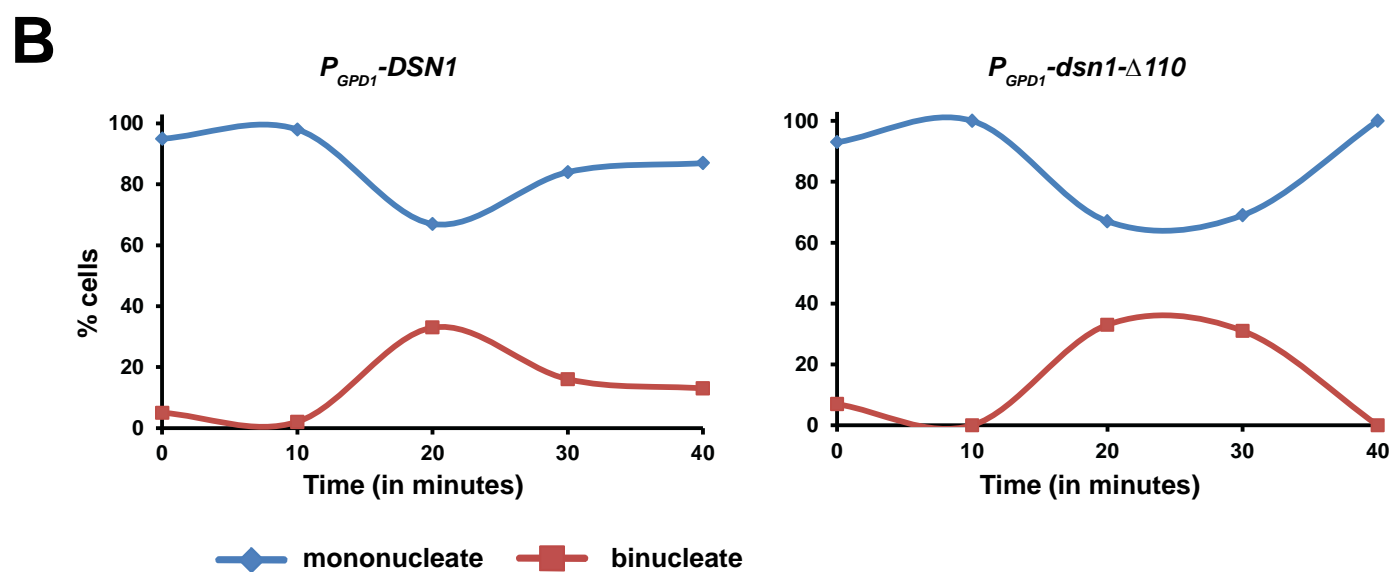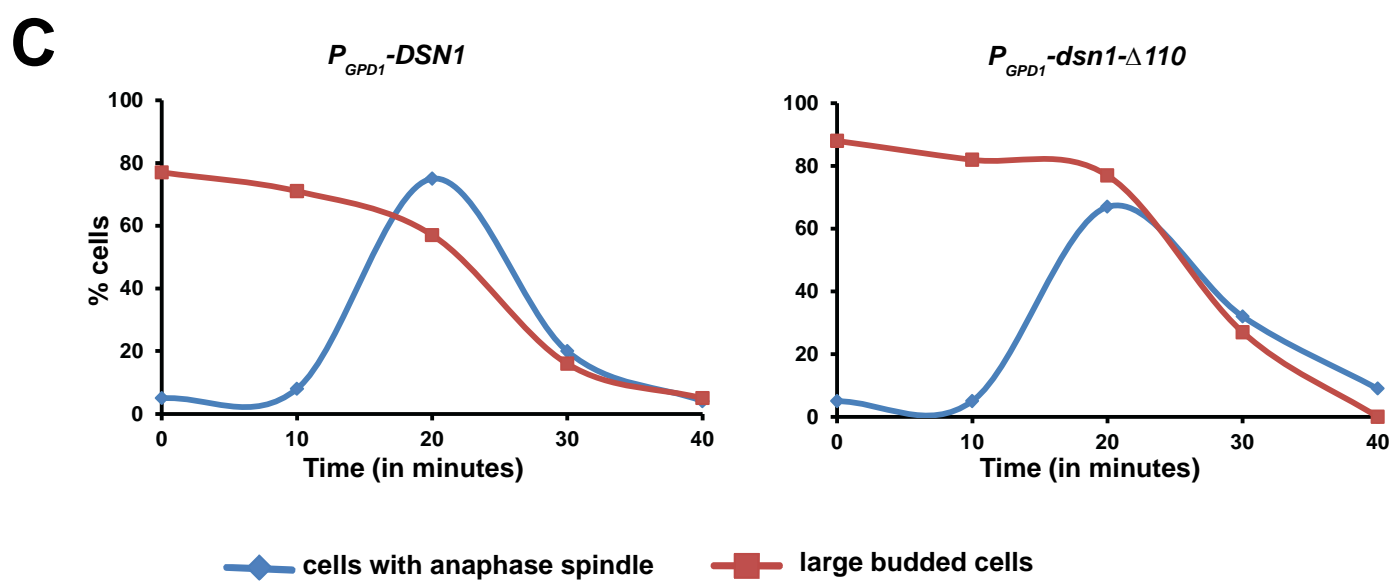

**Figure S5**

Supplement: Figure S5 — The Csm1-Interaction Domain of Dsn1 is required for association of Csm1/Lrs4 complex during mitotic anaphase. NDC10-ha6 LRS4-myc9 PMET3-CDC20 cells containing either PGPD1-DSN1 or PGPD1-dsn1-Δ110 were arrested in metaphase by addition of methionine to the growth medium. Cells were then released into anaphase by transferring them to a growth medium lacking methionine. A) Chromosome spreads were prepared and stained with anti-myc and anti-HA antibodies and DNA was visualized by staining with DAPI. The percentage of nuclei displaying i) >50% co-localization of Ndc10 and Lrs4 foci (green bar) ii) <50% co-localization of Ndc10 and Lrs4 foci (red bar) iii) No co-localization (blue bar) was measured. Representative images of nuclei belonging to three categories are depicted. B) Kinetics of nuclear division (scored by DAPI staining) is presented. C) Kinetics of anaphase spindle (spindle length>3 µm) assembly/disassembly and budding index are presented. (PDF) [file pgen.1003610.s005.pdf]

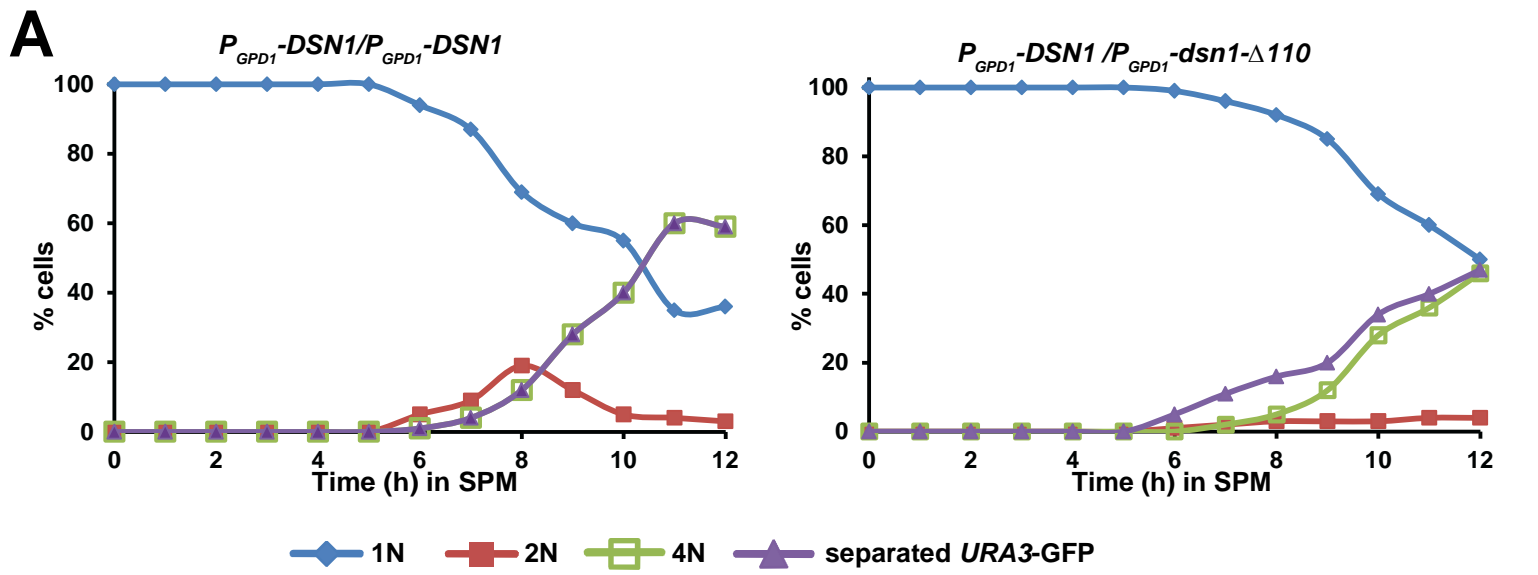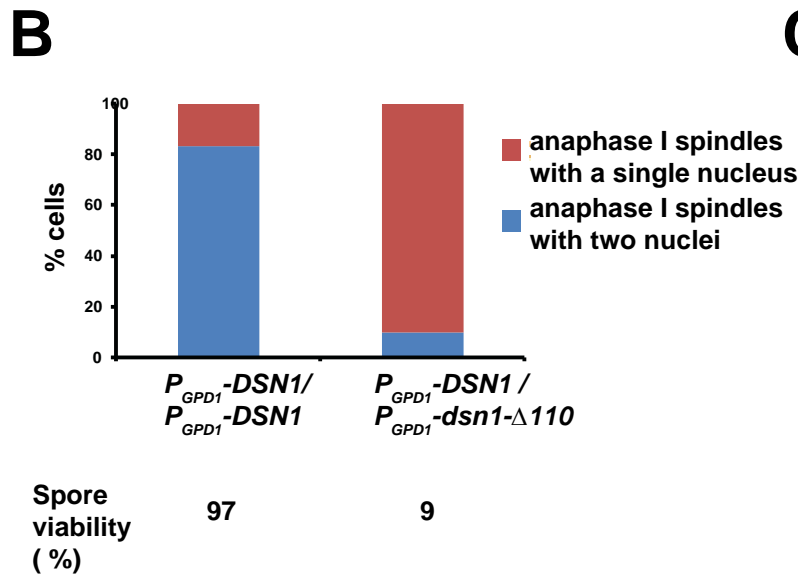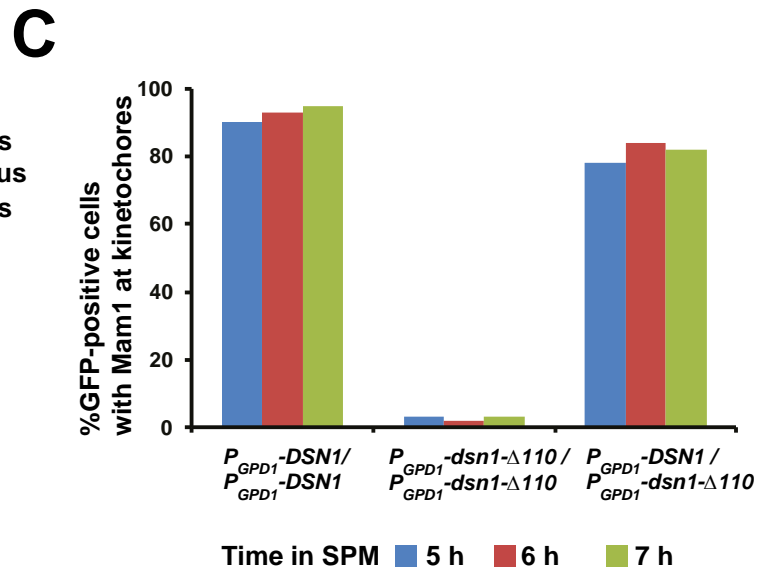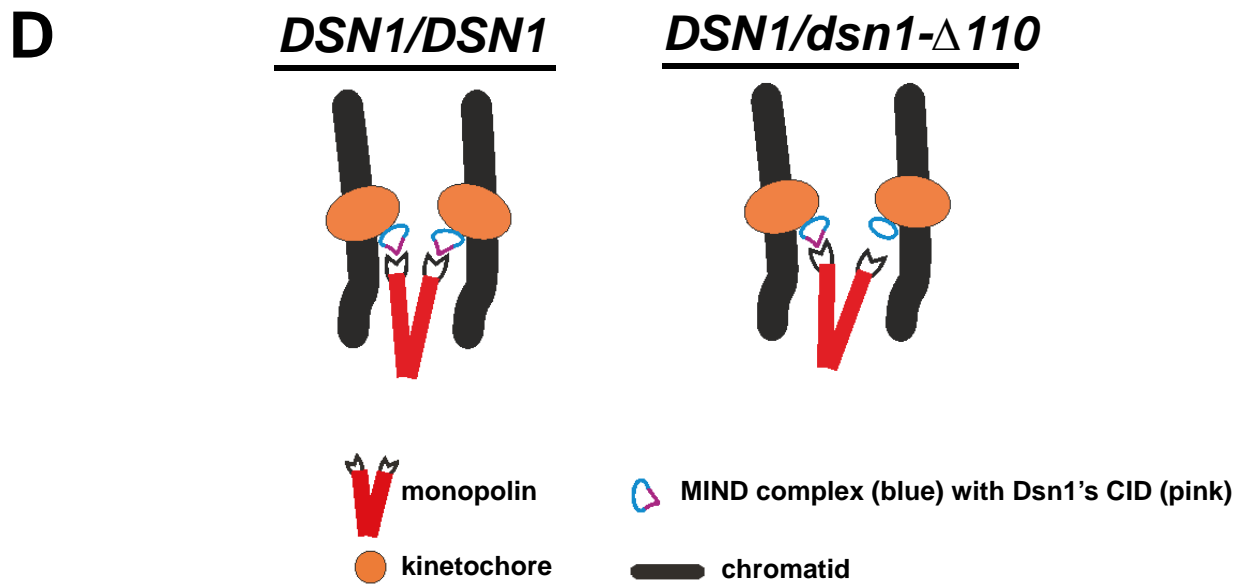

Figure S6

Supplement: Figure S6 — Expression of a dsn1 mutant lacking the Csm1-Interaction Domain dominantly interferes with monopolar attachment. A) Analysis of nuclear division and separation of URA3-GFP sequences during meiosis in PGPD1-DSN1/PGPD1-DSN1 and PGPD1-DSN1/PGPD1-dsn1-Δ110 cells expressing Pds1-myc18. Note that PGPD1-DSN1/PGPD1-dsn1-Δ110 cells fail to undergo the first meiotic nuclear division as shown by the absence of binucleates (2N) and separate URA3 dots before the appearance of tetranucleates. B) Percentage of anaphase I cells (Pds1 negative and one bipolar spindle) with divided or undivided nuclei. Spore viabilities of the two strains were determined (n = 100) and are indicated below their genotypes. C) MAM1-GFP MTW1-RFP PCLB2-CDC20 cells expressing either Dsn1 or Dsn1-Δ110 or Dsn1 and Dsn1-Δ110, were induced to enter meiosis by transferring them to SPM. Mam1 association with kinetochores was measured using GFP fixation assay after 5, 6 and 7 hours into SPM. D) An explanation why Dsn1 lacking the CID has a dominant negative effect on monopolar attachment. Kinetochores (brown) are assembled at centromeres on sister chromatids (black). Monopolin (in red) co-orients sister kinetochores in wild type cells by interacting with the CID in Dsn1, a part of the MIND complex (in blue). In cells expressing both Dsn1 and Dsn1-Δ110, monopolins fail to crosslink kinetochores as one of the two sister kinetochores lacks a monopolin-binding site (in pink). (PDF) [file pgen.1003610.s006.pdf]

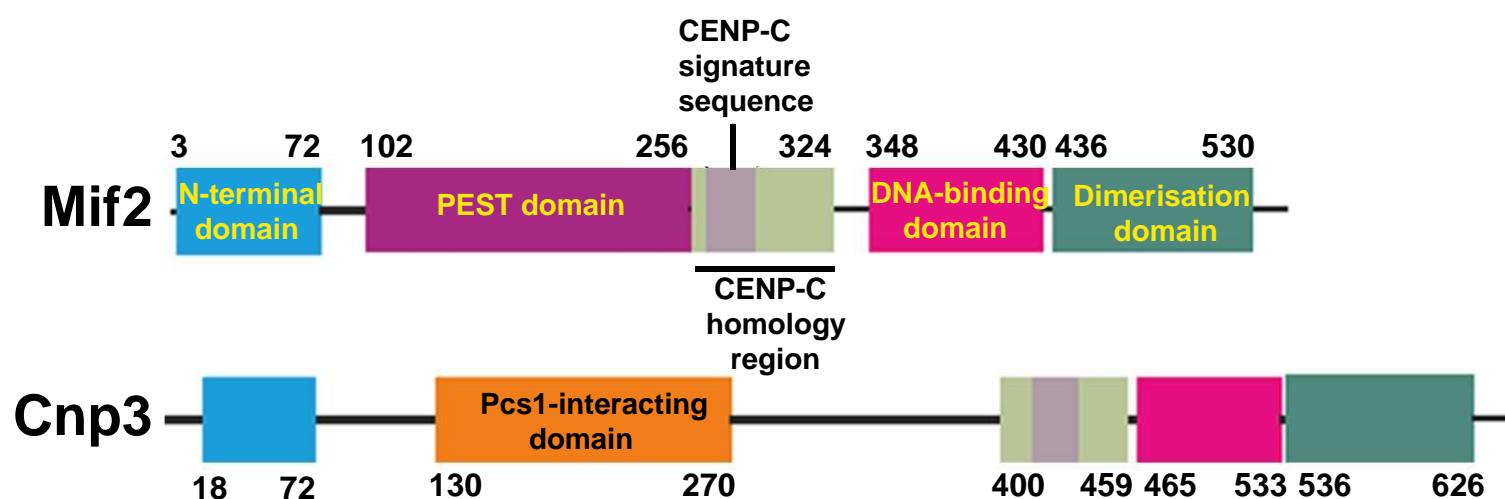

**Figure S7**

Supplement: Figure S7 — The Pcs1-interacting domain in fission yeast Cnp3 is not conserved in budding yeast Mif2. The positions of the N-terminal domain, CENP-C homology domain (containing the CENP-C signature sequence), DNA-binding domain and dimerization domain in Mif2 and Cnp3 are indicated. Mif2 lacks a region with sequence similarity to the Pcs1-interacting domain in Cnp3. (PDF) [file pgen.1003610.s007.pdf]
