# Supplementary material for: Monopolin Subunit Csm1 Associates with MIND Complex to Establish Monopolar Attachment of Sister Kinetochores at Meiosis I
Source: PLoS Genet. 2013 Jul 4;9(7):e1003610. doi: 10.1371/journal.pgen.1003610 (PMC3701701; doi:10.1371/journal.pgen.1003610)
Supplement: Table S1 — All yeast strains are derivatives of SK1 and have the following markers, unless otherwise stated. ho::LYS2/ho::LYS2, ura3/ura3, leu2::hisG/leu2::hisG, trp1::hisG/trp1::hisG, his3::hisG/his3::hisG, lys2/lys2. Markers are homozygous in diploid strains unless otherwise stated. (PDF) [file pgen.1003610.s008.pdf]

Supplementary Table S1

**Strain number**	**Genotype**	**Used in Figure**
1971	SK1 *MATa /MATα cep3::CEP3-RFP::NatMX6 mam1::MAM1-GFP::KlTRP1 cdc20::*P_{CLB2}*-CDC20::KanMX6*	1A
3105	SK1 *MATa /MATα cep3::CEP3-RFP::NatMX6 mam1::MAM1-GFP::KlTRP1 cdc20::*P_{CLB2}*-CDC20::KanMX6 lrs4::HIS3MX6*	1A
1974	SK1 *MATa /MATα cep3:CEP3-RFP::NatMX6 mam1:MAM1-GFP::KlTRP1 cdc20::*P_{CLB2}*-CDC20::KanMX6 ctf19:HIS3MX6*	1A
1964	SK1 *MATa /MATα cep3:CEP3-RFP::NatMX6 mam1:MAM1-yeGFP::KlTRP1 cdc20:*P_{CLB2}*-CDC20::KanMX6 mcm21::HphMX6*	1A
2016	SK1 *MATa /MATα cep3::CEP3-RFP::NatMX6 mam1::MAM1-GFP::KlTRP1 cdc20::*P_{CLB2}*-CDC20::KanMX6 mtw1::*P_{CLB2}*-MTW1::HIS3MX6*	1A
1977	SK1 *MATa /MATα cep3::CEP3-RFP::NatMX6 mam1::MAM1-GFP::KlTRP1 cdc20::*P_{CLB2}*-CDC20 dsn1:*P_{CLB2}*-DSN1::HIS3MX6*	1A
2981	*SK1 MATa /MATα cep3::CEP3-RFP::NatMX6 mam1:MAM1-GFP::KlTRP1 cdc20::*P_{CLB2}*-*	1A

	CDC20::KanMX6 mif2::P$_{CLB2}$-MIF2::HIS3MX6	
1837	*SK1 MATa /MATα mtw1::MTW1-RFP::HphMX6 mam1::MAM1-GFP::KlTRP1 cdc20::P$_{CLB2}$-CDC20::KanMX6*	1A –C , S1 and S4
1899	*SK1 MATa /MATα mtw1::MTW1-RFP::HphMX6 mam1::MAM1-GFP::KlTRP1 cdc20::P$_{CLB2}$-CDC20::KanMX6 spc24::P$_{CLB2}$-SPC24::HIS3MX6*	1A and S1
3200	*SK1 MATa /MATα mtw1::MTW1-RFP::HphMX6 mam1::MAM1-GFP::KlTRP1 cdc20::P$_{CLB2}$-CDC20::KanMX6 spc24::P$_{CLB2}$-ASK1::HIS3MX6*	1A and S1
3107	*SK1 MATa /MATα mtw1::MTW1-RFP::HphMX6 ndc10::NDC10-GFP::KlTRP1 cdc20::P$_{CLB2}$-CDC20::KanMX6 mif2:P$_{CLB2}$-MIF2::HIS3MX6*	1D
3109	*SK1 MATa /MATα mtw1::MTW1-RFP::HphMX6 ndc10::NDC10-GFP::KlTRP1 cdc20::P$_{CLB2}$-CDC20::KanMX6*	1D
1956	SK1 *MATa /MATα cep3::CEP3-RFP::NatMX6 mam1::MAM1-GFP::KlTRP1 cdc20::P$_{CLB2}$-CDC20::KanMX6 ctf3::HphMX6*	Table 1
1961	SK1 *MATa /MATα cep3::CEP3-RFP::NatMX6 mam1::MAM1-GFP::KlTRP1 cdc20::P$_{CLB2}$-CDC20::KanMX6 mcm22::HphMX6*	Table 1
1980	SK1 *MATa /MATα cep3::CEP3-RFP::NatMX6 mam1::MAM1-GFP::KlTRP1 cdc20::P$_{CLB2}$-*	Table 1

	CDC20::KanMX6 iml3::HphMX6	
2163	SK1 *MATa /MATα cep3::CEP3-RFP::NatMX6 mam1::MAM1-GFP::KlTRP1 cdc20::*P_{CLB2}*-CDC20::KanMX6 chl4::HphMX6*	Table 1
1966	SK1 *MATa /MATα cep3::CEP3-RFP::NatMX6 mam1::MAM1-GFP::KlTRP1 cdc20::*P_{CLB2}*-CDC20::KanMX6 nkp1::HphMX6*	Table 1
1968	SK1 *MATa /MATα cep3::CEP3-RFP::NatMX6 mam1::MAM1-GFP::KlTRP1 cdc20::*P_{CLB2}*-CDC20::KanMX6 nkp2::HphMX6*	Table 1
1958	SK1 *MATa /MATα cep3::CEP3-RFP::NatMX6 mam1::MAM1-GFP::KlTRP1 cdc20::*P_{CLB2}*-CDC20::KanMX6 mcm16::HphMX6*	Table 1
1910	SK1 *MATa /MATα mtw1::MTW1-RFP::NatMX6 mam1::MAM1-GFP::KlTRP1 cdc20::*P_{CLB2}*-CDC20::KanMX6 lrs4::HphMX6*	Table1
2129	AH109*; MATa trp1-901, leu2-3, 112, ura3-52, his3-200 gal4Δ, gal80Δ LYS2 : : GAL1UAS–Gal1TATA–His3, GAL2UAS–Gal2TATA–Ade2 URA3 : : MEL1UAS–Mel1TATAAUR1-C MEL1 pGBKT7 pGADT7*	2B
2130	*AH109; MATa trp1-901, leu2-3, 112, ura3-52 his3-200, gal4Δ, gal80Δ LYS2 : : GAL1UAS–Gal1TATA–*	2B

	His3, GAL2UAS–Gal2TATA–Ade2 URA3 : : MEL1UAS–Mel1TATAAUR1-C MEL1 pGBKT7 pGADT7-CSM1	
2862	*AH109; MATa trp1-901 leu2-3, 112 ura3-52 his3-200 gal4Δ gal80Δ LYS2 : : GAL1UAS–Gal1TATA–His3 GAL2UAS–Gal2TATA–Ade2 URA3 : : MEL1UAS–Mel1TATAAUR1-C MEL1 pGBKT7-DSN1 pGADT7-CSM1*	2B
2863	*AH109; MATa trp1-901 leu2-3, 112 ura3-52 his3-200 gal4Δ gal80Δ LYS2 : : GAL1UAS–Gal1TATA–His3 GAL2UAS–Gal2TATA–Ade2 URA3 : : MEL1UAS–Mel1TATAAUR1-C MEL1 pGBKT7-DSN1 pGADT7*	2B
2864	*AH109; MATa trp1-901 leu2-3, 112 ura3-52 his3-200 gal4Δ gal80Δ LYS2 : : GAL1UAS–Gal1TATA–His3 GAL2UAS–Gal2TATA–Ade2 URA3 : : MEL1UAS–Mel1TATAAUR1-C MEL1 pGBKT7-DSN1(1-220) pGADT7-CSM1*	2B
2865	*AH109; MATa trp1-901 leu2-3, 112 ura3-52 his3-200 gal4Δ gal80Δ LYS2 : :GAL1UAS–Gal1TATA–His3 GAL2UAS–Gal2TATA–Ade2 URA3 : : MEL1UAS–Mel1TATAAUR1-C MEL1 pGBKT7-DSN1(1-220B2A) pGADT7-CSM1*	2B
2866	*AH109; MATa trp1-901 leu2-3, 112 ura3-52 his3-200 gal4Δ gal80Δ LYS2 : : GAL1UAS–Gal1TATA–His3*	2B

	GAL2UAS–Gal2TATA–Ade2 URA3 : : MEL1UAS–Mel1TATAAUR1-C MEL1 pGBKT7-DSN1(1-220B3A) pGADT7-CSM1	
2054	*AH109; MATa trp1-901 leu2-3, 112 ura3-52 his3-200 gal4Δ gal80Δ LYS2 : :GAL1UAS–Gal1TATA–His3 GAL2UAS–Gal2TATA–Ade2 URA3 : : MEL1UAS–Mel1TATAAUR1-C MEL1 pGBKT7-DSN1(1-220B1A) pGADT7-CSM1*	2B
2956	*SK1 MATα csm1::CSM1-myc9::KlTRP1 dsn1::*P_{GPD1}*-DSN1-pk6::HIS3MX6 cdc20::* P_{CLB2}*-CDC20::KanMX6*	2C and 2D
2958	*SK1 MATα csm1::CSM1-myc9::KlTRP1 dsn1::*P_{GPD1}*-dsn1-Δ110-pk6::HIS3MX6 cdc20::* P_{CLB2}*-CDC20::KanMX6*	2C
1018	*SK1 MATa /MATα spo11::KlTRP1 spo12::KanMX4*	3A
1019	*SK1 MATa /MATα spo11::KlTRP1 spo12::KanMX4 mam1::HIS3MX6*	3A
2500	*SK1 MATa /MATα spo11::KlTRP1 spo12::KanMX4 dsn1::*P_{CLB2}*-DSN1::HIS3MX6*	3A
3197	*SK1 MATa /MATα spo11::KlTRP1 spo12::KanMX4 dsn1::*P_{GPD1}*-DSN1::natNT2*	3A
3195	*SK1 MATa /MATα spo11::KlTRP1 spo12::KanMX4 dsn1::*P_{GPD1}*-dsn1-Δ110::natNT2*	3A

2826	*SK1 MATa/MATα leu2::P$_{URA3}$::tetR::GFP-LEU2 rec8::REC8-ha3::URA3 pds1::PDS1-myc18::KlTRP1 ura3:: tetOx224::URA3 dsn1::P$_{GPD1}$-dsn1-Δ110:: KanMX4*	3B
2835	*SK1 MATa/MATα leu2::P$_{URA3}$::tetR::GFP::LEU2 rec8::REC8-ha3::URA3 pds1::PDS1-myc18::KlTRP1 ura3::tetOx224::URA3 dsn1::P$_{GPD1}$-DSN1::KanMX4*	3B
2823	*SK1 MATa/MATα leu2::P$_{URA3}$::tetR::GFP-LEU2 rec8::REC8-ha3::URA3 pds1::PDS1-myc18::KlTRP1 dsn1:P$_{GPD1}$-DSN1::KanMX4 ura3/ura3:: tetOx224::URA3*	3C-3E , S3 and S6
2824	*SK1 MATa/MATα leu2::P$_{URA3}$::tetR::GFP-LEU2 rec8::REC8-ha3::URA3 pds1::PDS1-myc18::KlTRP1 dsn1:P$_{GPD1}$-dsn1-Δ110::KanMX4 ura3/ura3:: tetOx224::URA3*	3C-3E and S3
1006	*SK1 MATa/MATα leu2::P$_{URA3}$::tetR::GFP-LEU2 rec8::REC8-ha3::URA3 pds1::PDS1-myc18::KlTRP1 mam1::HIS3MX6 ura3/ura3:: tetOx224::URA3*	S3
1007	*SK1 MATa/MATα leu2::P$_{URA3}$::tetR::GFP-LEU2 rec8::REC8-ha3::URA3 pds1::PDS1-myc18::KlTRP1 ura3/ura3:: tetOx224::URA3*	S3
1813	*SK1 MATa leu2::P$_{URA3}$::tetR::GFP-LEU2 rec8::REC8-ha3::URA3 pds1::PDS1-myc18::KlTRP1*	S2A
2700	*SK1 MATa leu2::P$_{URA3}$::tetR::GFP-LEU2 rec8::REC8-ha3::URA3 pds1::PDS1-myc18::KlTRP1*	S2A

	dsn1::P$_{GPD1}$-DSN1::KanMX4	
2758	*SK1 MATa leu2::P$_{URA3}$::tetR::GFP-LEU2 rec8::REC8-ha3::URA3 pds1::PDS1-myc18::KlTRP1 dsn1::P$_{GPD1}$-dsn1-Δ110::KanMX4*	S2A
2094	*SK1 MATa leu2::P$_{URA3}$::tetR::GFP-LEU2 rec8::REC8-ha3::URA3 pds1::PDS1-myc18::KlTRP1 ura3:: tetOx224::URA3 ctf19:HIS3MX6*	S2A
1537	*MAT α CFIII (CEN3.L.YPH278) URA3 SUP11ade2-101*	S2B
1575	*MAT α CFIII (CEN3.L.YPH278) URA3 SUP11ade2-101rts1:natMX4*	S2B
1501	*MAT α CFIII (CEN3.L.YPH278) URA3 SUP11ade2-101lrs4:HIS3MX6*	S2B
3191	*MAT α CFIII (CEN3.L.YPH278) URA3 SUP11ade2-101 dsn1:P$_{GPD1}$-DSN1::KanMX4*	S2B
3192	*MAT α CFIII (CEN3.L.YPH278) URA3 SUP11ade2-101 dsn1::P$_{GPD1}$-dsn1-Δ110::KanMX4*	S2B
3138	*SK1 MATa/MATα leu2::P$_{URA3}$::tetR::GFP-LEU2 rec8::REC8-ha3::URA3 pds1::PDS1-myc18::KlTRP1 dsn1:P$_{GPD1}$-DSN1::KanMX4/dsn1::P$_{GPD1}$-dsn1-Δ110::KanMX4 ura3/ura3:: tetOx224::URA3*	S6
3055	*SK1 MATa/MATα P$_{URA3}$::tetR::GFP::LEU2 rec8::P$_{REC8}$-SCC1-ha3::LEU2-KanMX6 spo11::KlTRP1 pds1::PDS1-myc18::TRP1 ura3/ura3:: tetOx224-URA3 DSN1/dsn1:P$_{GPD1}$-*	4A and 4B

	DSN1::KanMX4	
2967	*SK1 MATa/MATα P_{URA3}::tetR::GFP::LEU2 rec8::P_{REC8}-SCC1-ha3::LEU2-KanMX6 spo11::KlTRP1 pds1::PDS1-myc18::TRP1 ura3/ura3:: tetOx224-URA3 DSN1/dsn1:P_{GPD1}-dsn1-Δ110::KanMX4*	4A and 4B
2854	*SK1 MATa /MATα mtw1::MTW1-RFP::HphMX6 mam1:MAM1-GFP::KlTRP1 cdc20::P_{CLB2}-CDC20::HIS3MX6 dsn1::P_{GPD1}-DSN1::KanMX4*	5A-C , S4 and S6C
2856	*SK1 MATa /MATα mtw1::MTW1-RFP::HphMX6 mam1:MAM1-GFP::KlTRP1 cdc20::P_{CLB2}-CDC20::HIS3MX6 dsn1::P_{GPD1}- dsn1-Δ110::KanMX4*	5A-C , S4 and S6C
2166	*SK1 MATa /MATα mtw1::MTW1-RFP::HphMX6 mam1:MAM1-GFP::KlTRP1 cdc20::P_{CLB2}-CDC20::HIS3MX6 dsn1::P_{GPD1}- dsn1-Δ110::KanMX4 /dsn1::P_{GPD1}-DSN1::KanMX4*	S6C
2959	*SK1 MATa /MATα csm1::CSM1-myc9::klTRP1 dsn1::KanMX4-P_{GPD1}-DSN1-pk6::HIS3MX6 cdc20::P_{CLB2}-CDC20-HIS3MX6*	5D
2957	*SK1 MATa /MATα csm1::CSM1-myc9::klTRP1 dsn1::KanMX4-P_{GPD1}-dsn1-Δ110-pk6::HIS3MX6 cdc20::P_{CLB2}-CDC20::HIS3MX6*	5D
3049	*SK1 MATα lrs4::LRS4-myc9::klTRP1 dsn1::KanMX4-*	S5

	P_{GPD1}-*dsn1-Δ110-pk6::HIS3MX6 ndc10:NDC10-ha6::HIS3MX6 cdc20::*P_{MET3}-*CDC20::LEU2*	
3050	*SK1 MATα lrs4::LRS4-myc9::klTRP1 dsn1::KanMX4-*P_{GPD1}-*DSN1-pk6::HisMX6 ndc10:NDC10-ha6::HIS3MX6 cdc20::*P_{MET3}-*CDC20::LEU2*	S5
